# Genetic-by-age interaction analyses on complex traits in UK Biobank and their potential to identify effects on longitudinal trait change

**DOI:** 10.1186/s13059-024-03439-9

**Published:** 2024-11-28

**Authors:** Thomas W. Winkler, Simon Wiegrebe, Janina M. Herold, Klaus J. Stark, Helmut Küchenhoff, Iris M. Heid

**Affiliations:** 1 Department of Genetic Epidemiology, University of Regensburg, Franz-Josef-Strauß-Allee 11, Regensburg, 93053 Germany; 2 Statistical Consulting Unit StaBLab, Department of Statistics, LMU Munich, Geschwister-Scholl-Platz 1, Munich, 80539 Germany

**Keywords:** GWAS, Genetic-by-age interaction, Longitudinal, Obesity, Lipids, Blood pressure, UK Biobank

## Abstract

**Background:**

Genome-wide association studies (GWAS) have identified thousands of loci for disease-related human traits in cross-sectional data. However, the impact of age on genetic effects is underacknowledged. Also, identifying genetic effects on longitudinal trait change has been hampered by small sample sizes for longitudinal data. Such effects on deteriorating trait levels over time or disease progression can be clinically relevant.

**Results:**

Under certain assumptions, we demonstrate analytically that genetic-by-age interaction observed in cross-sectional data can be indicative of genetic association on longitudinal trait change. We propose a 2-stage approach with genome-wide pre-screening for genetic-by-age interaction in cross-sectional data and testing identified variants for longitudinal change in independent longitudinal data. Within UK Biobank cross-sectional data, we analyze 8 complex traits (up to 370,000 individuals). We identify 44 genetic-by-age interactions (7 loci for obesity traits, 26 for pulse pressure, few to none for lipids). Our cross-trait view reveals trait-specificity regarding the proportion of loci with age-modulated effects, which is particularly high for pulse pressure. Testing the 44 variants in longitudinal data (up to 50,000 individuals), we observe significant effects on change for obesity traits (near *APOE*, *TMEM18*, *TFAP2B*) and pulse pressure (near *FBN1*, *IGFBP3*; known for implication in arterial stiffness processes).

**Conclusions:**

We provide analytical and empirical evidence that cross-sectional genetic-by-age interaction can help pinpoint longitudinal-change effects, when cross-sectional data surpasses longitudinal sample size. Our findings shed light on the distinction between traits that are impacted by age-dependent genetic effects and those that are not.

**Supplementary Information:**

The online version contains supplementary material available at 10.1186/s13059-024-03439-9.

## Background

Genome-wide association studies (GWAS) based on cross-sectional data have significantly enhanced the understanding of the genetic underpinning of complex diseases and disease-related traits by identifying thousands of genetic loci associated with diseases or traits [1]. However, they are limited in their ability to capture genetic effects on trait changes over time. Such effects indicate genetic susceptibility to deteriorating biomarker levels and even disease progression [2]. They can be clinically relevant and may inform personalized medicine approaches, allowing for the early identification of individuals at risk or the development of targeted interventions [2]. Thus recently, there has been a growing interest in longitudinal GWAS, as evidenced by longitudinal GWAS of biomarker trajectories in UK Biobank (UKB) [3], a longitudinal GWAS of BMI trajectories in UKB [4], or several longitudinal GWAS of kidney function from the CKDGen consortium [5] or from the Million Veterans Program [6]. However still, only few genetic effects on trait change over time have been identified. Identification of such effects has been hampered by relatively small sample sizes for longitudinal measurements.

We postulate a link between the genetics of trait change over time from longitudinal data with genetic-by-age interaction in cross-sectional data: we hypothesize that a genetic-by-age interaction effect estimated from cross-sectional data can serve as indicator of a genetic effect on longitudinal change under certain assumptions. Then, cross-sectional genetic-by-age interactions can reflect the genetic influence on how trait levels change as individuals age. Here, we propose a 2-stage approach to gain power in testing for genetic effects on longitudinal trait change. The approach comprises a first stage to select variants with significant genetic-by-age interaction in cross-sectional data and a second stage to test the selected variants for association with trait-change in longitudinal data. The 2-stage approach reduces the multiple testing burden in the longitudinal data by exploiting relatively large cross-sectional sample sizes, and thus power, in the first stage.

The aim of this research is to demonstrate, analytically and empirically, when the genetic-by-age interaction and the genetic effect on linear trait change are equivalent. We demonstrate that integrating cross-sectional genetic-by-age interactions can boost power to identify genetics of trait change in longitudinal GWAS. We apply our approach to the UKB cross-sectional and longitudinal data and aim to identify genetic-by-age interaction and longitudinal change loci for obesity, lipid, and blood pressure traits. We highlight traits for which genetic effects can change by age and demonstrate that genetics-by-age interactions have been underacknowledged.

## Results

### Limited availability of longitudinal data in UKB

Utilizing UKB data for European individuals, we set out to identify genetic-by-age interaction and genetic associations for trait change for eight complex traits: two obesity traits (weight and body mass index, BMI), three blood pressure traits (systolic blood pressure, SBP; diastolic blood pressure, DBP; pulse pressure, PP), and three lipid traits (HDL-cholesterol, HDL-C; LDL-cholesterol, LDL-C; triglycerides, TG). For obesity and blood pressure traits, two timepoints were available in up to ~ 50,000 individuals (Table 1). In contrast, lipid traits, two timepoints were available in only up to 15,000 individuals. For all traits, the number of individuals with longitudinal information was considerably less, about one fifth or 1/20th, compared to the respective cross-sectional sample size available at the baseline visit (> 380,000 individuals for all traits; Table 1, Additional file 1: Table S1). Clearly, power of a longitudinal GWAS to identify longitudinal change effects in this relatively small longitudinal data is limited.

**Table 1:** UKB cross-sectional and longitudinal sample sizes. Shown are the number of European individuals in UKB with baseline and longitudinal data available on each of eight complex traits. The cross-sectional data values shown are based on all individuals or limited to those that attended the baseline visit but none of the follow-up visits. The longitudinal data values shown are based on individuals with data available from baseline and at least one of follow-up visits (regular follow-up visit; for obesity and blood pressure measurements also from the imaging and the repeated imaging visit). The linear effect of age (beta Age) on the outcome (adjusted for sex and 5 PCs) and its explained variance (*R*^2^) is shown for the baseline data. The “change per year” values reflect annual change of the outcome per year and are derived from the difference of the outcome between two visits, divided by the time between the two visits (for individuals with more than two assessments, we have used the difference between repeated, imaging, and repeated imaging visit with the baseline assessment in descending order, i.e., preferably used the regular repeated visit). A detailed statistical description of various subsets of the data can be found in Additional file 1: Table S1. A detailed description of UKB data variables and trait transformations can be found in “Methods”

Outcome	Unit	Cross-sectional				Longitudinal			
		**Baseline**		**Baseline (excl. long.)**			**Baseline**	**Follow-up**	**Change**
		*N*	beta Age (*R*^2^)	*N*	Mean (SD)	*N*	Mean (SD)	Mean (SD)	Mean (SD)
Weight	kg	424,199	− 0.072 (0.1%)	371,541	78.4 (16.0)	52,658	77.3 (15.1)	76.7 (15.3)	− 0.084 (0.9)
BMI	kg/m^2^	424,044	0.027 (0.2%)	371,416	27.5 (4.82)	52,628	26.7 (4.34)	26.7 (4.49)	0.001 (0.32)
DBP	mmHg	388,409	0.19 (1.8%)	346,259	84.6 (11.2)	42,150	83.4 (11.0)	82.1 (11.1)	− 0.21 (1.58)
SBP	mmHg	388,400	0.78 (10.0%)	346,256	141.9 (20.7)	42,144	139.0 (19.8)	143.4 (20.8)	0.54 (2.76)
PP	mmHg	388,400	0.77 (18.9%)	346,256	57.2 (14.3)	42,144	55.7 (13.3)	61.3 (15.1)	0.75 (2.06)
HDL-ln	logn mg/dl	371,297	0.0015 (0.2%)	359,117	4.0 (0.26)	12,180	4.0 (0.26)	4.1 (0.26)	0.01 (0.04)
LDL	mg/dl	404,900	0.79 (3.2%)	389,885	148.2 (35.4)	15,015	147.7 (35.6)	151.2 (36.5)	0.94 (7.8)
TG-ln	logn mg/dl	405,326	0.0066 (1.1%)	390,270	4.9 (0.52)	15,056	4.9 (0.51)	4.9 (0.48)	0 (0.11)

We demonstrate the relationship between genetic-by-age interaction in cross-sectional data and genetic effects on annual change in longitudinal data in the following. We also investigate whether integrating genetic-by-age interaction searches in relatively large cross-sectional data can support the identification of genetic effects on annual change.

### Analytical relationship between cross-sectional genetic-by-age interaction and genetics of longitudinal change

An analysis to identify genetic-by-age interaction effects in cross-sectional data (i.e., one measurement per person) requires a regression model that includes a genetic-by-age interaction term:

$$Y={\beta }_{0}+{\beta }_{G}G+{\beta }_{Age}AGE+{\beta }_{GxAge}G\cdot AGE+{\beta }_{C}C+\varepsilon$$

Here, *Y* is the trait value of the individual in the cross-sectional data, *G* the allelic dosage, *AGE* is the age of the individual centered at the mean age of study individuals, and *C* is a matrix of further covariates. The estimated interaction effect size, ${\widehat{\beta }}_{GxAge}$, can be interpreted as annual change in the genetic effect on the trait when comparing individuals of different age.

In comparison, a typical GWAS on annual change of a trait can be conducted based on data with two trait measurements over time and a linear regression model:

$$\frac{{Y}_{2}-{Y}_{1}}{{t}_{2}-{t}_{1}}={\gamma }_{0}+{\gamma }_{G}G+{\gamma }_{C}C+\epsilon$$

Here, *Y*_1_ and *Y*_2_ are the trait values of an individual at timepoint *t*_1_ and *t*_2_, respectively, *G* is the allele dosage of a genetic variant, and *C* a matrix of covariates at timepoint *t*_1_. The estimated genetic effect size, ${\widehat{\gamma }}_{G}$, can be interpreted as genetic effect on annual trait change.

**Fig. 1:**
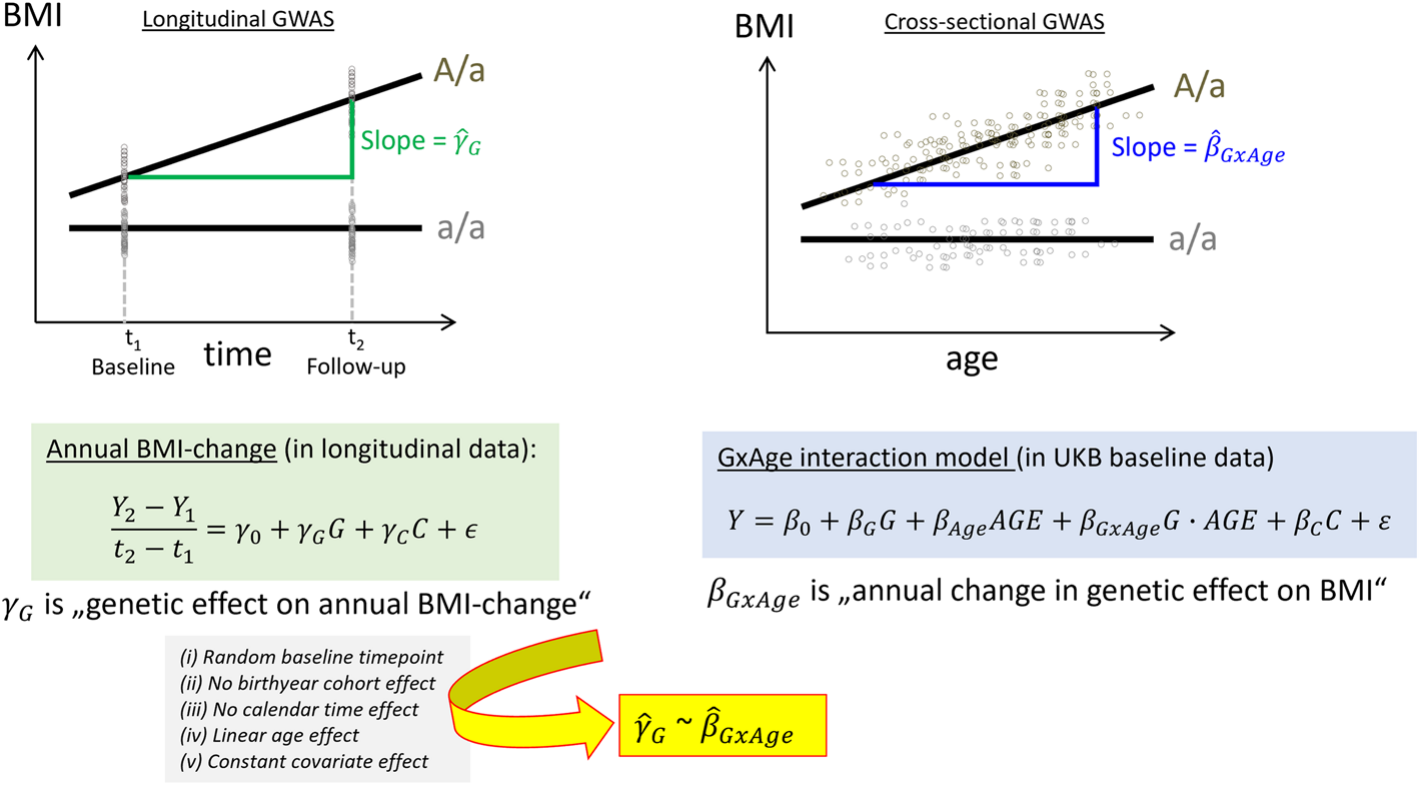
Relationship between longitudinal and cross-sectional genetic-by-age interaction GWAS models. The figure illustrates the similarity between genetic effects on annual trait change (estimated using longitudinal data from two timepoints) and genetic-by-age interaction effects (estimated from cross-sectional data) and states the assumptions for the equivalence. The figure demonstrates the genotype effects on the example of BMI for individuals with homozygous a/a (no time/age-dependency, no BMI effect) compared to individuals with heterozygous A/a genotypes (time/age-dependent BMI effect)

Under the assumption that *t*_1_ is a random timepoint and does not mark an intervention (observational data), that there is no calendar time effect on *Y*, no birth cohort effect on *G* and *Y*, that the trait changes linearly over age, and that the covariate effects are independent of age, we demonstrate the equivalence ${\widehat{\gamma }}_{G}$ ~ ${\widehat{\beta }}_{GxAge}$ (Fig. 1, Additional file 2: Note S1). This implies that a genetic-by-age interaction effect on *Y* can reflect the genetic effect on annual trait-change when assumptions are met. Consequently, a genome-wide search for genetic-by-age interaction should identify genetic associations with annual trait-change. However, it is also important to acknowledge that genetic-by-age interaction effect size estimates can potentially be confounded by various aspects, such as unmodeled covariate-by-age or genetic-by-covariate interaction terms, or complex LD structure [7, 8]. Since it may be difficult in practice to rule out potential departure from the assumptions or confounding, we also seek, for genetic variants with identified genetic-by-age interaction, a validation of the variant’s association with change in independent longitudinal data—which is especially important when the primary aim is to identify robust genetic effects on longitudinal change.

We postulate that conducting a GWAS testing for genetic-by-age interactions can be employed to increase power to identify genetic effects on longitudinal trait change when the cross-sectional sample size outnumbers the longitudinal sample size available.

### Screening for genetic-by-age interaction improves power to identify genetic effects on annual trait change

**Fig. 2:**
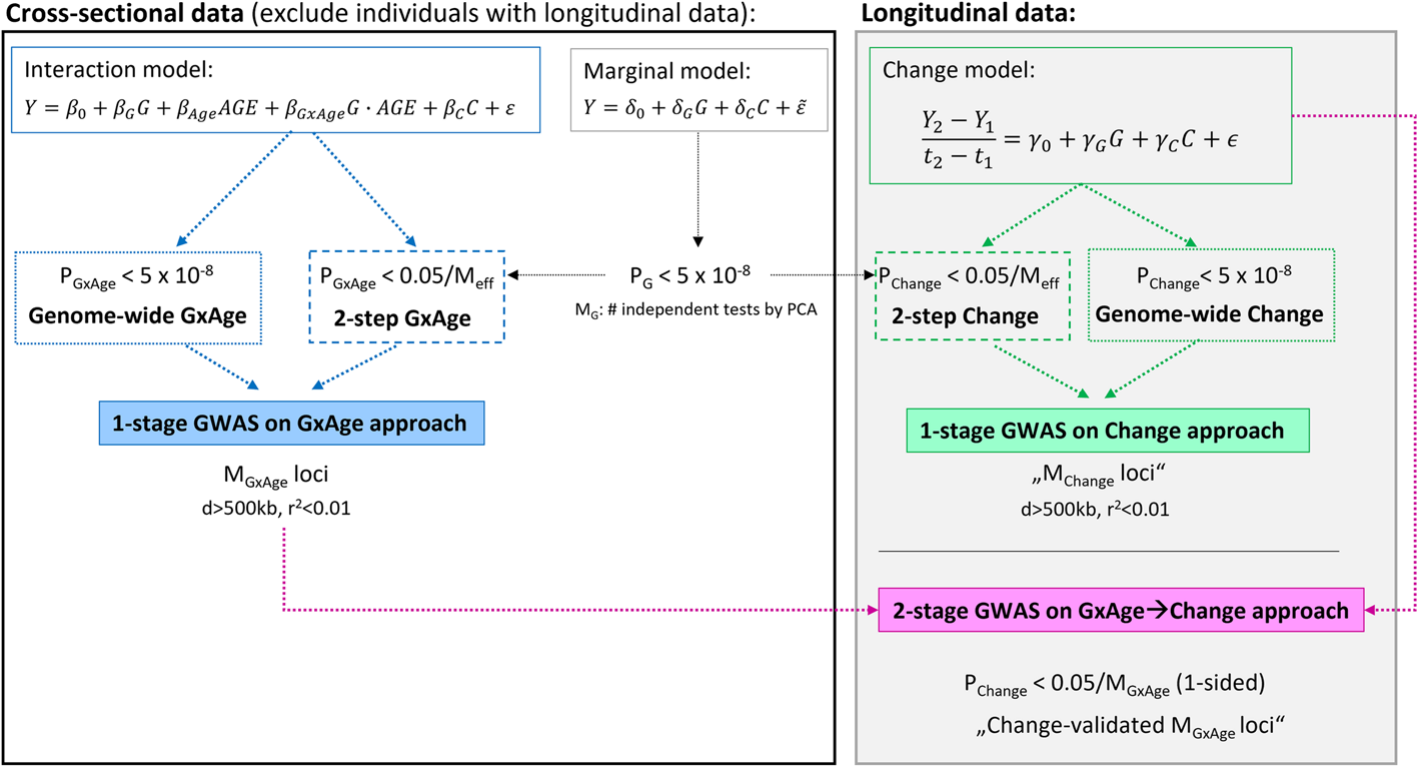
Approaches to identify longitudinal change effects. Shown is the workflow of three approaches considered to identify annual change effects: (i) the 1-stage GWAS on GxAge approach in cross-sectional data (blue; assuming equivalence of genetic-by-age interaction and annual change effects; involves genetic-by-age interaction testing at genome-wide significance, *P*_GxAge_ < 5 × 10^−8^, and a 2-step approach focused on variants with genome-wide significant marginal effects, *P* < 5 × 10^−8^; then *P*_GxAge_ < 0.05/*M*_eff_, corrected for the number of effective tests among marginally associated variants); (ii) the 2-stage GWAS on GxAgeChange approach that includes additional validation for annual change effects in independent longitudinal data (magenta); and (iii) the 1-stage GWAS on change approach in longitudinal data (green; involves annual change association testing at genome-wide significance, *P*_Change_ < 5 × 10.^−8^, and a 2-step approach focused on variants with genome-wide significant marginal effects, *P*_GxAge_ < 0.05/*M*_eff_)

To investigate whether screening for genetic-by-age interaction in cross-sectional data can help to identify genetic effects on trait change, we compared the power of three approaches (workflow of approaches shown in Fig. 2, “Methods”): (i) A GWAS for genetic-by-age interaction in cross-sectional sample data (“1-stage GxAge” approach). (ii) A GWAS for genetic-by-age interaction in cross-sectional data (same as (i)) followed by validation of identified variants for annual change effects in independent longitudinal data (“2-stage GxAgeChange” approach). The two approaches are compared to (iii): A GWAS for trait change in the longitudinal data alone (“1-stage Change” approach). Power was computed based on analytical power formulae shown in Additional file 2: Note S2 (“Methods”).

**Fig. 3:**
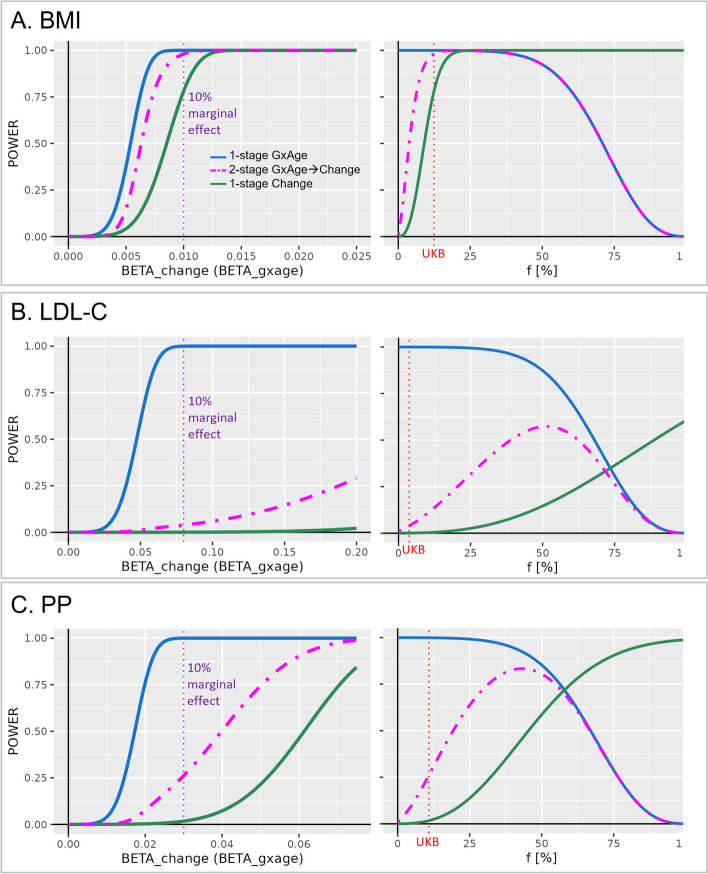
Power to identify genetic-by-age interaction and longitudinal change effects. Shown are power curves for genetic-by-age interaction and annual change effects on **A** BMI, **B** LDL-C, and **C** PP. Power is shown for the three approaches: the 1-stage GWAS on GxAge approach in cross-sectional data (blue), the 2-stage GWAS on GxAgeChange approach that includes additional validation for annual change effects in independent longitudinal data (magenta), and the 1-stage GWAS on change approach in longitudinal data (green). For each trait, the left panel shows power over varying effect size (varied from zero to 25% of a median marginal genetic effect on the trait; purple vertical dotted line denotes 10% of the medium marginal effect) while keeping cross-sectional and longitudinal sample sizes constant at UKB sample sizes for the trait (Table 1). The right panel shows power of varying longitudinal-to-total sample size ratios (*f*), while keeping total sample size constant at the UKB trait sample size (Table 1; *N*_long_ = f*N_total_; *N*_cross_ = *N*_total_ − *N*_long_; the red vertical dotted line denotes *f* as given in UKB for the respective trait) and keeping the genetic effect constant at the 10% medium marginal effect size (purple dotted line in the left panel). Power was calculated for an allele frequency of 30%, based on analytical formulas given in Additional file 2: Note S2 and assumptions given in “Methods.” Power computations for the remaining traits are shown in Additional file 2: Fig. S1

We first compared the power of the three approaches for varying effect sizes and for sample size configurations as in UKB (cross sectional *N* > 345,000 and independent longitudinal *N* < 55,000; proportion of longitudinal data among total sample size < 15%). We observed a substantial improvement in power to identify genetic effects on trait change by the two approaches involving “genetic-by-age interaction testing” compared to the “1-stage Change” approach (left panels in Fig. 3, Additional file 2: Fig. S1). For example, assuming a realistic genetic effect on annual trait change that is equal to 10% of a medium marginal effect for the trait (i.e., the genetic effect is modulated per year by 10%), we observe > 90% power for the “1-stage GxAge” approach across all traits (Table 2). Power of the two approaches involving “change” for this 10% effect was adequate for weight and BMI (> 77%) but extensively attenuated for the other traits (< 27%, Table 2). This pattern was confirmed by calculations of minimum annual change effect size detectable at 80% power for the three approaches (Table 2): While all approaches capture realistic annual change effects (modulation per year ≤ 10% of a medium marginal genetic effect) for weight and BMI, only the “1-stage GxAge” approach captures such realistic annual change effects for the other traits (Table 2). The minimum effect sizes detectable by the two approaches involving “change” were unrealistically high (e.g., 0.46 (mg/dl)/year/allele for the “1-stage Change” approach on LDL-C, which refers to modulation per year that is equivalent to 58% of a medium marginal genetic effect).

**Table 2:** Power and minimum detectable effect size for the three approaches. Shown are assumptions and results for analytical power computations for the “1-stage GxAge” approach in cross-sectional data (excluding individuals with longitudinal data), the “2-stage GWAS on GxAgeChange” approach that includes additional validation for annual change effects in independent longitudinal data, and the “1-stage Change” approach in longitudinal data. Power is shown for realistic annual change (genetic-by-age interaction) effect sizes that were fixed at 10% of the median marginal effect for the respective trait (as observed in UKB; data not shown; power > 80% marked in bold). Minimum annual change (genetic-by-age interaction) effect sizes detectable at 80% power were obtained and compared to the median marginal effects (realistic annual change effect sizes that are ≤ 10% of the median marginal effect were marked in bold). Power and minimum effect sizes were calculated for realistic UKB sample size configurations for the respective trait (proportion of longitudinal data < 15%). We assumed an allele frequency of 30% and based the computation on analytical formulae (“Methods,” Additional file 2: Note S2)

Trait	Config	*N*_Cross_	*N*_Long_	SD_Y_	SD_change_	Annual change effect = 10% medium marginal effect							Min. detectable annual change effect (%medium marginal effect)		
						**Medium *b***_**Marginal**_	***b***_**Change**_** (*b***_**GxAge**_**)**	***R***^**2**^_**GxAge**_	***R***^**2**^_**Change**_	**Pwr 1-stage GxAge**	**Pwr 2-stage GxAgeChange**	**Pwr 1-stage Change**	**1-stage GxAge**	**2-stage GxAgeChange**	**1-stage Change**
Weight	UKB	371,541	52,658	15.1	0.9	0.3	0.03	0.009%	0.047%	**0.99**	**0.98**	**0.87**	**0.024 (8%)**	**0.025 (8%)**	**0.029 (10%)**
BMI	UKB	371,416	52,628	4.34	0.32	0.1	0.01	0.013%	0.041%	**1.00**	**0.98**	0.78	**0.0069 (7%)**	**0.0078 (8%)**	**0.010 (10%)**
DBP	UKB	346,259	42,150	10.95	1.58	0.2	0.02	0.008%	0.007%	**0.92**	0.17	0.01	**0.018 (9%)**	0.041 (20%)	0.057 (29%)
SBP	UKB	346,256	42,144	19.77	2.76	0.4	0.04	0.010%	0.009%	**0.99**	0.25	0.02	**0.032 (8%)**	0.071 (18%)	0.098 (25%)
PP	UKB	346,256	42,144	13.28	2.06	0.3	0.03	0.012%	0.009%	**1.00**	0.26	0.02	**0.022 (7%)**	0.053 (18%)	0.073 (24%)
HDL_ln	UKB	359,117	12,180	0.26	0.04	0.005	5.0E − 04	0.009%	0.007%	**0.97**	0.05	0.00	**4.2E − 04 (8%)**	0.0019 (38%)	0.0026 (52%)
LDL-C	UKB	389,885	15,015	35.58	7.84	0.8	0.08	0.012%	0.004%	**1.00**	0.04	0.00	**0.055 (7%)**	0.34 (42%)	0.46 (58%)
TG_ln	UKB	390,270	15,056	0.51	0.11	0.01	0.001	0.009%	0.003%	**0.99**	0.03	0.00	**7.9E − 04 (8%)**	0.0047 (47%)	0.0065 (65%)

This UKB scenario with the proportion of longitudinal data relative to total sample size (*f*) being only up to 15% is rather unique. To generalize to studies with higher *f*, we compared the power of the three approaches for varying *f* while keeping the total sample size constant (*N*_total_ = *N*_cross_ + *N*_long_, trait-specific) and keeping the annual change effects constant (at 10% of a medium marginal genetic effect). We expected a “kipping point” for *f* where the “1-stage GxAge” approach lost superiority in power against the “1-stage Change” approach. We observed trait-dependent kipping points at *f* = 25%, 75%, and 65% for BMI, LDL-C, or pulse pressure (Fig. 3, Additional file 2: Fig. S1). For UKB sample size, power of the “1-stage Change” approach was deprecated compared to genetic-by-age interaction approaches for all traits (red dotted lines in Fig. 3 and Additional file 2: Fig. S1).

Interestingly, and a bit counterintuitive at first glance, equal sample size for cross-sectional and longitudinal data (*f* = 0.5, *N*_cross_ = *N*_long_ = *N*, number of measurements *N* and 2*N, respectively) yielded superior power for the cross-sectional “1-stage GxAge” approach compared to longitudinal “1-stage Change” approach for blood pressure and lipid traits (e.g., for pulse pressure 85% versus 59%, for LDL-C 82% versus 12%), but reduced power for obesity traits (e.g., for BMI 92% versus 99%; Fig. 3, Additional file 2: Fig. S1). This trait-dependent pattern was observed despite, across traits, the same significance level, similar sample size, and similar genetic-by-age interaction effect size relative to the phenotypic variance (i.e., similar outcome variance explained by the genetic-by-age interaction, *R*^2^_GxAge_ of ~ 0.01%, Table 2). However, a driving factor for power in longitudinal analyses is also the variance of annual outcome change explained by the genetic effect (*R*^2^_Change_). Indeed, we observed a smaller *R*^2^_Change_ for pulse pressure and LDL-C (0.009% and 0.004%, respectively) compared to BMI and weight (*R*^2^_Change_ ~ 0.04% for both traits; Table 2).

To explore the reasons for these trait-dependent power differences further, we compared power of a genetic-by-age interaction test and an annual change association test in a more controlled scenario (fixed parameters of *N*_cross_ = *N*_long_ = 200 K, alpha = 5 × 10^−8^, allele frequency AF = 0.3, beta_GxAge_ = beta_Change_). We demonstrate that the power of the genetic-by-age interaction test is larger than power of the annual change test, as long as $\left[2*Var\left(Age\right)\right]>\left[{age}_{diff}^{2}/(1-{r}_{Y1,Y2})\right]$ (Additional file 2: Note S3). This can be interpreted intuitively: (i) the larger the age range and thus the age variance in the cross-sectional data, the larger the power of the genetic-by-age interaction test, (ii) the longer the follow-up time (age_diff_) in the longitudinal data and the closer *r*_*Y*1,*Y*2_ to 1 (i.e., little technical measurement error or intra-individual variability), the larger the power of the annual change test. This explains the observed trait-specific pattern (Additional file 1: Table S2): the power of the annual change test compared to the genetic-by-age interaction test (i) is superior for BMI due to relatively large follow-up time and high correlation (age_diff_ ~ 7.5 years, *r*_*Y*1,*Y*2_ ~ 0.91), (ii) inferior for pulse pressure due to the lower correlation (age_diff_ ~ 7.5 years, *r*_*Y*1,*Y*2_ ~ 0.65), and (iii) near zero for LDL-C due to shorter follow-up time and lower correlation (age_diff_ ~ 4.3 years, *r*_*Y*1,*Y*2_ ~ 0.62). Thus, the genetic-by-age interaction test can be particularly helpful for traits where measurement error or intra-individual variability is high and when follow-up length is short.

In summary, our GWAS involving genetic-by-age interaction testing in cross-sectional data has reasonable power to identify realistic genetic-by-age interaction effects for all traits; the 2-stage approach with additional validation of the genetic effect on annual change in independent longitudinal data has substantially larger power than the GWAS for change in longitudinal data alone. The power of the GWAS for change alone is limited in this UKB data due to the short follow-up time. We thus applied the “1-stage GxAge” and the “2-stage GxAgeChange” approach to the eight traits in UKB and describe the results in the following section.

### Genome-wide search identifies 44 significant genetic-by-age interaction

**Fig. 4:**
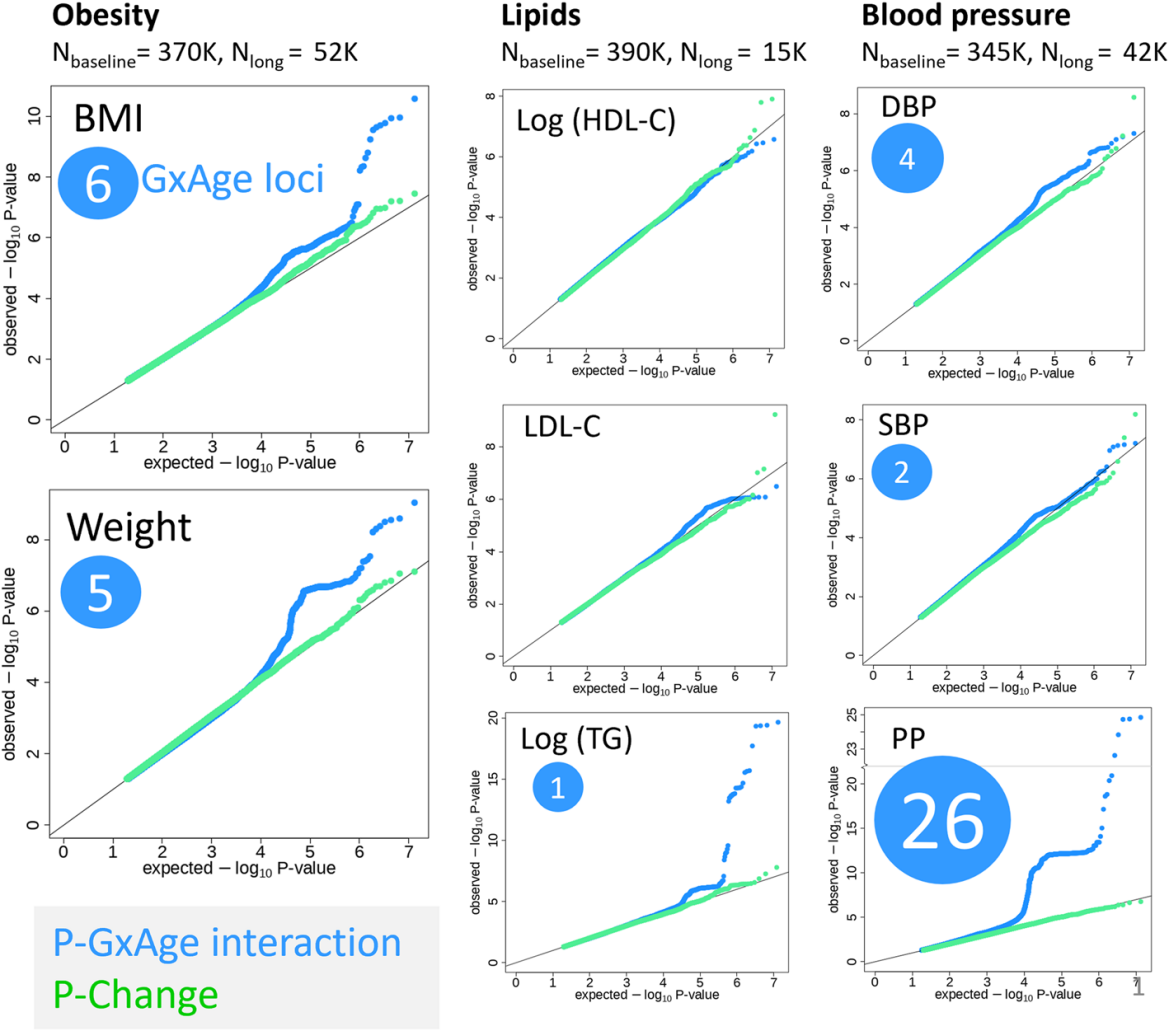
Cross-sectional genetic-by-age interaction GWAS and longitudinal GWAS results for 8 complex traits. For the eight traits, shown are the quantile–quantile (QQ) plots for the genetic-by-age interaction *P* values (blue; testing in cross-sectional UKB data excluding individuals with longitudinal data available; approx. sample size shown in figure) and for the association *P* values for annual change (green; testing in longitudinal UKB data; approx. sample size shown in figure). Indicated in blue circles are the number of significant genetic-by-age interaction loci (*d* > 500 kb and *r*^2^ < 0.01) identified in the cross-sectional data (*P*_GxAge_ < 5 × 10^−8^, or by the 2-step approach focused on marginal effects, *P* < 5 × 10^−8^; then *P*_GxAge_ < 0.05/*M*_eff_). Green are QQ plots for the annual trait change association *P* values from the respective longitudinal UKB data

**Fig. 5:**
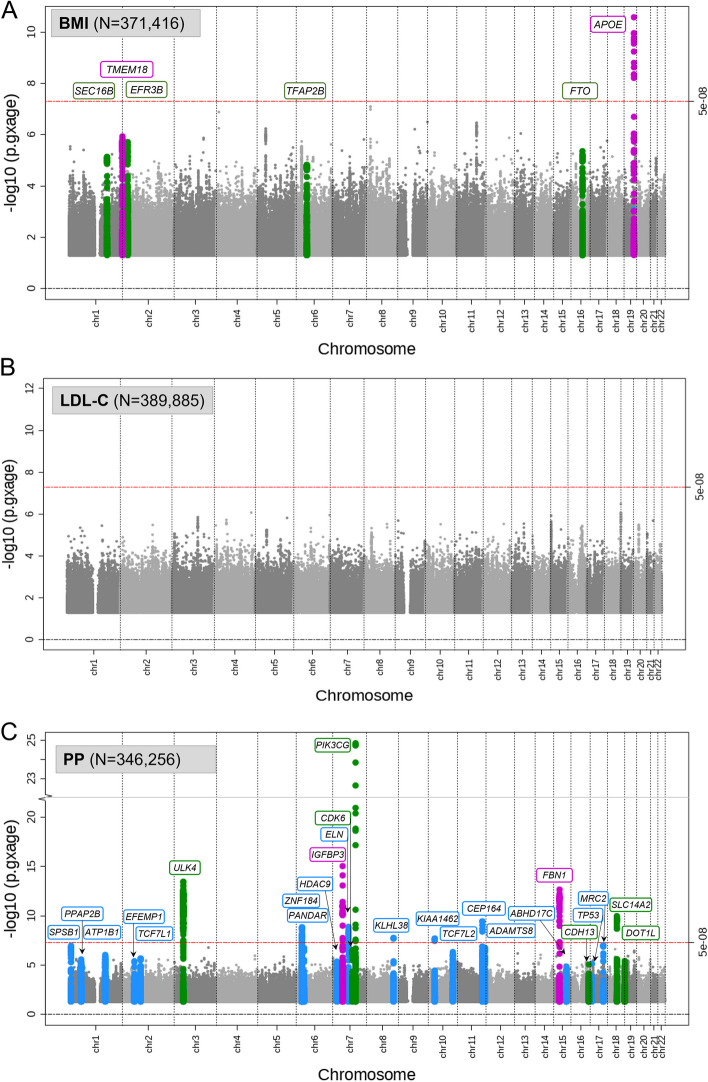
Manhattan plots of genetic-by-age interaction for BMI, LDL-C, and pulse pressure. The figure shows the genome-wide Manhattan plots of genetic-by-age interaction *P* values for **A** BMI, **B** LDL-C, and **C** pulse pressure. These are based on cross-sectional data from UKB excluding individuals with longitudinal data (cross-sectional *N* > 340,000). Significant genetic-by-age interaction loci (*P*_GxAge_ < 5 × 10^−8^; or 2-step significant: marginal *P* < 5 × 10^−^.^8^ and *P*_GxAge_ < 0.05/*M*_eff_) are colored in blue, green, and magenta. The different coloring indicates association of the index variant with annual trait-change in independent longitudinal data from UKB (longitudinal *N* up to 52,000): blue indicates lack of annual trait-change association (1-sided *P*_Change_ ≥ 0.05), green indicates nominal-significant, directionally consistent annual trait-change effects (1-sided *P*_Change_ < 0.05), and magenta indicates Bonferroni-corrected significant, directionally consistent annual trait-change effects (1-sided *P*_Change_ < 0.05/*M*_GxAge_, corrected for the number of significant genetic-by-age interaction loci per trait)

To identify genetic-by-age interactions for the eight traits, we analyzed the cross-sectional baseline data of UKB excluding individuals with longitudinal data for the respective trait (“Methods,” Additional file 1: Table S3). Across the eight traits, we identified a total of 44 significant genetic-by-age interaction effects (*P*_GxAge_ < 5 × 10^−8^, or *P*_GxAge_ < 0.05/*M*_eff_ among variants with marginal *P* < 5 × 10^−8^; *d* > 500 kb, *r*^2^ < 0.01 to identify independent index variants per trait; Fig. 4, Additional file 1: Table S4). The 44 genetic-by-age interaction index variants mostly pertained to three traits: 11 interactions were identified for weight or BMI (located at 7 loci; including loci near *APOE*, *TMEM18*, and *FTO*) and 26 for pulse pressure (26 loci; Fig. 5, Additional file 2: Fig. S2). Fewer loci were identified for triglycerides (1 locus, near *APOE*), diastolic (4 loci; near *IGFBP3*, *SDCCAG8*, *FGF5*, and *LILRP2*), and systolic blood pressure (2 loci, near *PIK3CG* and *ABHD17C*). No significant genetic-by-age interactions were identified for HDL-cholesterol and for LDL-cholesterol.

As expected, given the correlation between some of the traits (data not shown), there was overlap of identified loci between traits (i.e., same, or correlated index variants were identified by two or more traits). For example, the *APOE* index variant rs429359 was identified for significant genetic-by-age interaction for BMI, weight, and triglycerides (Additional file 1: Table S4). Overall, the 44 genetic-by-age interaction index variants pertained to 36 independent loci (*d* > 500 kb, *r*^2^ < 0.01 between index variants, Additional file 2: Fig. S3). Gene prioritization based on DEPICT [9] and FUMA [10] highlighted 83 genes at these loci (Additional file 1: Table S5). Clearest evidence was found for 11 genes that were prioritized by both methods at genetic-by-age interaction loci for pulse pressure: *CDH13*, *CDKN1A*, *DOT1L*, *EFEMP1*, *FBN1*, *FBXO32*, *IGFBP3*, *KIAA1462*, *MRC2*, *SPSB1*, *TCF7L1*.

The identified genetic-by-age interaction effects mostly overlapped with marginal effect loci for the respective traits, i.e., 40 of the 44 genetic-by-age interaction index variants showed genome-wide significant marginal effects on the respective trait here (marginal *P* < 5 × 10^−8^, Additional file 1: Table S4, Additional file 2: Fig. S4). This is in line with the idea that interaction effects are among marginal effects when the trait-deteriorating allele of the marginal effect is the trait-deteriorating allele independent of age (no cross-over). Vice versa, starting from trait-specific genome-wide significant marginal effect lead variants (marginal *P* < 5 × 10^−8^, *d* > 500 kb, Additional file 1: Table S6), we observed a significant intensification of effects with increasing age for systolic blood pressure and pulse pressure, and a significant attenuation of effects for weight, BMI, and LDL-cholesterol (enrichment *P*_binom_ < 0.05/8, Bonferroni-corrected for 8 traits, Table 3). On average, the yearly modulation of genetic effect size ranged between 3.2 and 4.8% of the median marginal effect size across traits. The strongest enrichment was observed for pulse pressure where intensified effects were observed for 101 (56.4%) of the 179 marginally associated region lead variants. This trait-specific enrichment provides the degree of the trait’s genetics that is age-dependent and its direction.

**Table 3:** Proportion and directionality of genetic-by-age interaction effects among marginal associated regions. The table shows the number of marginal associated regions (*d* < 500 kb, *P*_Marginal_ < 5 × 10^−8^) for the eight traits identified in UKB (using cross-sectional baseline data excluding any individual with longitudinal data available) and the median marginal effect sizes among the region lead variants (trait unit per allele). For the marginal lead variants with nominal significant genetic-by-age interaction effects (*P*_GxAge_ < 0.05), the table shows their proportions among all marginal lead variants, their directions (i.e., whether marginal effects are intensified or attenuated with age), the directional enrichment (2-sided binomial test for enrichment comparing intensified vs. attenuated variants), and the median genetic-by-age interaction effects. Significant directional enrichments are marked in bold (binomial *P* < 0.05/8)

Trait	*N*_Nolong_	Number of marginal regions	Median *b*_Marginal_	Number of nominal sig. GxAge regions (% among marginal regions)			Directional enrichment *P*_binom_	Median *b*_GxAge_ (% median *b*_Marginal_)
				**All**	**Intensified**	**Attenuated**		
Weight	371,541	248	0.29	42 (16.9%)	3 (1.2%)	**39 (15.7%)**	**5.6E − 09**	0.011 (3.8%)
BMI	371,416	212	0.093	32 (15.1%)	1 (0.5%)	**31 (14.6%)**	**1.5E − 08**	0.0036 (3.8%)
DBP	346,259	202	0.22	36 (17.8%)	16 (7.9%)	20 (9.9%)	0.62	0.0089 (4.0%)
SBP	346,256	182	0.40	34 (18.7%)	**29 (15.9%)**	5 (2.8%)	**3.9E − 05**	0.015 (3.7%)
PP	346,256	179	0.28	102 (57.0**%**)	**101 (56.4%)**	1 (0.6%)	**4.1E − 29**	0.014 (4.8%)
HDL-ln	359,117	236	0.0051	22 (9.3%)	15 (6.4%)	7 (3.0%)	0.13	1.6E − 04 (3.2%)
LDL	389,885	218	0.77	28 (12.8%)	5 (2.3%)	**23 (10.6%)**	**9.1E − 04**	0.025 (3.2%)
TG-ln	390,270	211	0.011	20 (9.5%)	5 (2.4%)	15 (7.1%)	0.041	4.1E − 04 (3.6%)

In summary, we identified a total of 44 genetic-by-age interactions mapping to 36 independent loci across the eight traits with the most interaction loci being identified for BMI and pulse pressure and only very few for lipid traits.

### Sensitivity analyses for identified 44 genetic-by-age interaction variants

We explored potential confounding by birth cohort effect for the identified genetic-by-age interaction effects on the respective trait. While this can be explored by adjusting for birthyear, genetic-by-birthyear, or age-by-birthyear interaction in theory, this is often impossible in practice when age and birthyear are highly correlated (collinearity). This is the case in UKB (*r* = − 0.99; “Methods”). For the 44 variants identified with genetic-by-age interaction, we attempted adjusting for birthyear and found no impact on genetic-by-age interaction effects, but this was not surprising as this adjustment just reflected the adjustment for age; we also evaluated the genetic variant association with birthyear, but again this was expected to be equivalent to the association with age (Additional file 1: Table S7).

Confounding of the genetic-by-age interaction by birthyear as well as age-dependent selection or survival would imply an association of the genetic variant with age. Among the 44 identified variants, we found only the *APOE* variant rs429358 associated with age (*P*_G__Age_ = 4.6 × 10^−5^, Additional file 1: Table S7). Since the rs429358 allele that is less frequent in older age (C allele) is well-known for increasing risk of Alzheimer [11] and early mortality [12], it is perceivable that this variant’s association with age is explained by decreased study participation among Alzheimer patients (selection) or decreased survival rather than a birth cohort effect. While the observed *APOE* genetic-by-age interaction on BMI, weight, and triglycerides is adjusted for age (i.e., removing age-dependent selection or survival effects at least in part), there might be residual confounding. Our sensitivity analyses to identify genetic variant association with age were reasonably powered (> 95% to identify *APOE* effect on age of 0.12 years/allele; minimal detectable effect size at 80% power = 0.09 years per allele, explains ~ 0.005% of the age variance; *N* = 350,000, alpha = 0.05/44, AF = 30%).

### Longitudinal validation of genetic-by-age interaction loci for annual change effects

To preclude departure from assumptions, we sought validation of the 44 identified variants for their association on trait change in independent longitudinal data from the UKB (longitudinal *N* up to 52,628, Table 1).

Among the 44 genetic-by-age interaction variants, we observed an enrichment of 19 nominal-significant and directionally consistent genetic effects on annual change of the respective trait in the longitudinal data (1-sided *P*_change_ < 0.05; enrichment *P* = 8.0 × 10^−14^, Table 4, Additional file 1: Table S4). These included seven variants that were significant for trait change at a Bonferroni-corrected alpha level (1-sided *P*_change_ < 0.05/*M*_GxAge_, Bonferroni-corrected for a trait-level number of genetic-by-age interaction loci, *M*_GxAge_; Table 4, Additional file 1: Table S4): two for BMI-change (near *APOE* and *TMEM18*; longitudinal *N* = 52,628), three for weight-change (near *APOE*, *TMEM18*, and *TFAP2B*; longitudinal *N* = 52,658), and two for pulse pressure change (near *FBN1* and *IGFBP3*; longitudinal *N* = 42,144). Only the *APOE* variant for BMI and weight would have been identified by a 1-stage GWAS on change in the longitudinal data alone (*APOE*: weight *P*_Change_ = 4.5 × 10^−8^ and BMI *P*_Change_ = 3.2 × 10^−7^, i.e., 2-step significant among marginal associated variants, Additional file 1: Table S4). The other five change associations were identified only after pre-screening for genetic-by-age interaction in the cross-sectional data but would have been missed in an analysis using longitudinal data only. Some lack of validation for other genetic-by-age interaction loci might be attributed to power (Table 2, Fig. 3, Additional file 2: Fig. S1): for example, we would require ~ 340,000 individuals with longitudinal data on pulse pressure change to successfully validate (at 80% power) a median observed genetic-by-age interaction effect of 0.021 mmHg/year/allele.

**Table 4:** Validation of genetic-by-age interaction loci for annual change effects. The 19 variants were identified for significant genetic-by-age interaction in cross-sectional data (UKB, excluding individuals with longitudinal data available; *P*_GxAge_ < 5 × 10^−8^ or *P*_GxAge_ < 0.05/*M*_eff_ for variants with marginal *P* < 5 × 10^−8^; Bonferroni-corrected at trait-level for the number of effective tests estimated by PCA, *M*_eff_) and further showed nominal significant genetic effects on annual trait change in independent longitudinal data (at 1-sided *P*_change_ < 0.05). Seven of the 19 change associations were significant at a trait-specific Bonferroni-corrected alpha level (shown in bold; corrected for the number of genetic-by-age interaction loci for the trait, *M*_GxAge_, all at 1-sided *P*_change_ < 0.05/*M*_GxAge_). The effect directions were aligned to marginally trait-increasing alleles

Trait	rsid	chr	pos	Gene	ea	oa	Cross-sectional							Longitudinal			
							**eaf**	***b***_**marginal**_	***P***_**marginal**_	***b***_**gxage**_	**se**_**gxage**_	***P***_**gxage**_	***N***_**cross**_	***b***_**change**_	**se**_**change**_	***P***_**change**_	***N***_**change**_
Obesity traits																	
BMI	rs429358	19	45,411,941	***APOE***	T	C	0.84	0.12	7.0E − 12	0.012	0.002	2.6E − 11	371,416	0.014	0.003	**1.6E − 07**	52,628
BMI	rs71392529	2	638,838	***TMEM18***	G	GTTT	0.79	0.23	7.2E − 47	− 0.008	0.002	1.2E − 06	371,416	− 0.006	0.002	**0.0082**	52,628
BMI	rs16880854	6	50,904,881	*TFAP2B*	G	A	0.17	0.20	4.9E − 31	− 0.008	0.002	1.5E − 05	371,416	− 0.006	0.003	0.011	52,628
BMI	rs564667	2	25,310,860	*EFR3B*	T	A	0.57	0.12	1.3E − 21	− 0.006	0.001	1.9E − 06	371,416	− 0.004	0.002	0.012	52,628
BMI	rs630372	1	177,885,762	*SEC16B*	G	A	0.23	0.20	8.2E − 41	− 0.007	0.002	7.3E − 06	371,416	− 0.004	0.002	0.040	52,628
BMI	rs9937521	16	53,799,296	*FTO*	C	T	0.42	0.35	2.5E − 163	− 0.006	0.001	4.4E − 06	371,416	− 0.003	0.002	0.045	52,628
Weight	rs429358	19	45,411,941	***APOE***	T	C	0.84	0.32	1.7E − 09	0.033	0.005	9.5E − 10	371,541	0.041	0.008	**4.5E − 08**	52,658
Weight	rs71392529	2	638,838	***TMEM18***	G	GTTT	0.79	0.70	3.1E − 49	− 0.027	0.005	3.6E − 08	371,541	− 0.019	0.007	**0.0025**	52,658
Weight	rs62405439	6	50,847,486	***TFAP2B***	C	A	0.17	0.57	2.5E − 29	− 0.026	0.005	6.7E − 07	371,541	− 0.018	0.007	**0.0065**	52,658
Weight	rs9937521	16	53,799,296	*FTO*	C	T	0.42	0.98	4.7E − 143	− 0.020	0.004	4.2E − 07	371,541	− 0.011	0.006	0.022	52,658
Weight	rs36162392	6	26,569,135	*HMGN4*	T	C	0.12	0.32	4.6E − 08	− 0.028	0.006	4.7E − 06	371,541	− 0.014	0.008	0.041	52,658
Pulse pressure																	
PP	rs2015637	15	48,716,853	***FBN1***	T	C	0.90	0.55	7.4E − 20	0.043	0.006	2.2E − 13	346,256	0.076	0.024	**6.8E − 04**	42,144
PP	rs11977526	7	46,008,110	***IGFBP3***	G	A	0.60	0.47	2.6E − 37	0.029	0.004	9.1E − 16	346,256	0.042	0.014	**0.0019**	42,144
PP	rs7236548	18	43,097,750	*SLC14A2*	A	C	0.18	0.50	5.7E − 26	0.030	0.005	1.1E − 10	346,256	0.052	0.018	0.0022	42,144
PP	rs8102624	19	2,161,443	*DOT1L*	A	G	0.07	0.68	3.6E − 22	0.032	0.007	3.8E − 06	346,256	0.073	0.027	0.0039	42,144
PP	rs60814640	7	92,286,918	*CDK6*	A	G	0.66	0.38	3.3E − 23	0.019	0.004	2.9E − 07	346,256	0.031	0.015	0.018	42,144
PP	rs2392929	7	106,414,069	*PIK3CG*	G	T	0.20	0.97	2.1E − 100	0.047	0.004	1.4E − 25	346,256	0.036	0.018	0.020	42,144
PP	rs7500448	16	83,045,790	*CDH13*	A	G	0.75	0.45	3.1E − 26	0.018	0.004	8.8E − 06	346,256	0.031	0.016	0.028	42,144
PP	rs190379045	3	41,882,697	*ULK4*	G	A	0.88	0.71	3.7E − 33	0.043	0.006	3.6E − 14	346,256	0.039	0.023	0.044	42,144

**Fig. 6:**
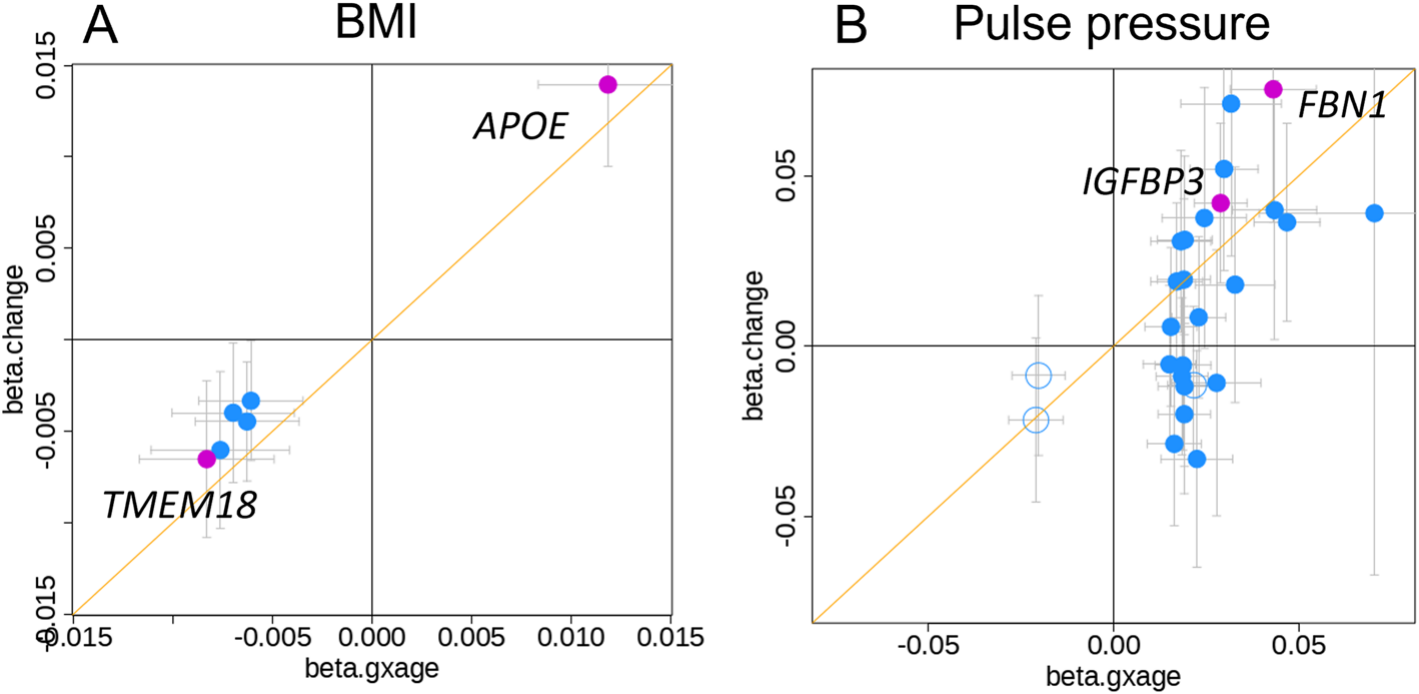
Comparison of genetic-by-age interaction and annual change effect sizes. Shown is a comparison of genetic-by-age interaction effect sizes with annual change effect sizes for the change-validated loci **A** six BMI-loci and **B** 26 pulse pressure loci. The loci displayed significant genetic-by-age interaction in cross-sectional data (UKB, excluding individuals with longitudinal data available; *P*_GxAge_ < 5 × 10^−8^ or *P*_GxAge_ < 0.05/*M*_eff_ for variants with marginal *P* < 5 × 10^−8^; Bonferroni-corrected at trait-level for the number of effective tests estimated by PCA, *M*_eff_). Loci that further showed significant genetic effects on annual trait change in independent longitudinal data are colored magenta (all at 1-sided *P*_change_ < 0.05/*M*_GxAge_; Bonferroni-corrected at trait-level for the number of genetic-by-age interaction loci, *M*_GxAge_). The effect directions of the variants were aligned to marginally trait-increasing alleles. Solid circles indicate variants with genome-wide significant marginal effects (marginal *P* < 5 × 10.^−8^)

Given the mathematical similarity between genetic-by-age interaction and genetic effects on change, we were interested in whether this similarity was also observable empirically in terms of effect sizes. We compared beta-estimates of the genetic-by-age interaction and the genetic effect on change for the 44 identified variants: as expected, the effect sizes of the seven variants validated for trait change at a Bonferroni-corrected alpha level were highly comparable (including the *APOE* variant), as were the effect sizes for the 12 additional variants with nominal significance (Fig. 6). Larger longitudinal data would have been required to show the equivalence of effect sizes for all 44 variants empirically.

In summary, our approach to identify variants with genetic-by-age interaction in cross-sectional data with validation for effects on change in independent longitudinal data successfully identified annual change loci that would have been missed by a search in longitudinal data alone. Observed annual change effect sizes aligned well with the respective genetic-by-age interaction effect sizes. Yet, power to identify annual change in UKB alone was hampered by the relatively low longitudinal sample size in UKB.

### Genetic-by-age interactions highlight trait-specific biological aging processes

Among the eight traits studied, most genetic-by-age interactions were identified for obesity (BMI or weight; 7 loci) and pulse pressure (26 loci). Only few were identified for other blood pressure traits or lipids.

**Fig. 7:**
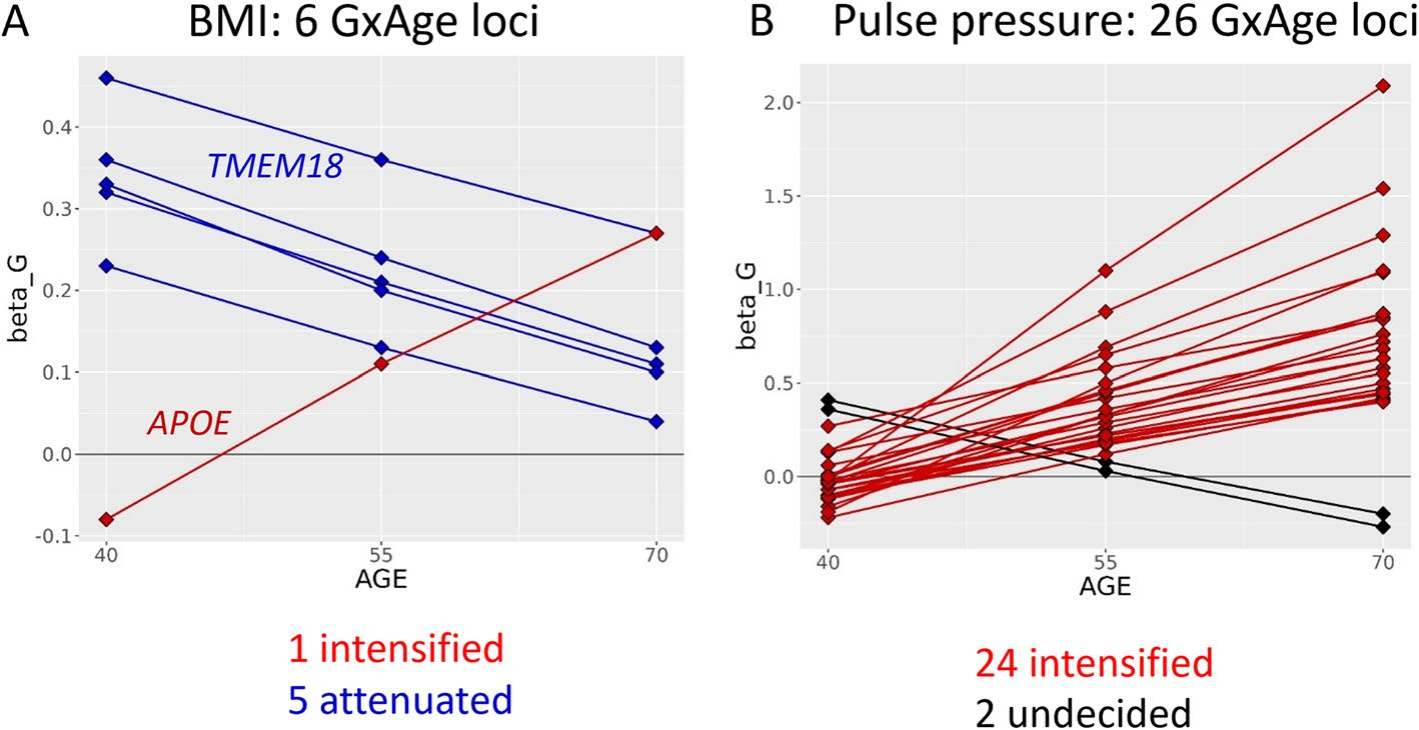
Direction of genetic-by-age interactions for BMI and pulse pressure. For the variants with significant genetic-by-age interaction, the figures show the genetic effect estimates on **A** BMI and **B** pulse pressure, at 40, 55, and 70 years of age. The age-specific genetic effects were based on the observed genetic main and genetic-by-age interaction effect sizes from the genetic-by-age interaction regression model and by substituting ages 40, 55, and 70 into the model. The effect directions were aligned to trait-increasing alleles

The extent of genetic-by-age interaction might tell something about underlying mechanisms: the lack of genetic-by-age interactions for lipid traits in this data means that genetic effects on lipids remain relatively constant during the age of 40 to 70 years, which suggests that the genetic variant causes a genotype-dependent offset early on that remains constant in middle-aged adulthood. To visualize the age-dependency of genetic effects on BMI and pulse pressure that we identified with genetic-by-age interaction, we estimated the genetic effects at 40, 55, and 70 years of age (Fig. 7, Additional file 1: Table S4).

For BMI, the genetic effects were attenuated with increased age towards a zero genetic effect for 5 of the 6 variants (all except *APOE*; enrichment *P* = 0.22, Fig. 7A). As stated above, the *APOE* variant, rs429358, was associated with age, which might be explained by age-dependent selection or survival effects due to this variant’s association with Alzheimer’s disease and early mortality.

**Fig. 8:**
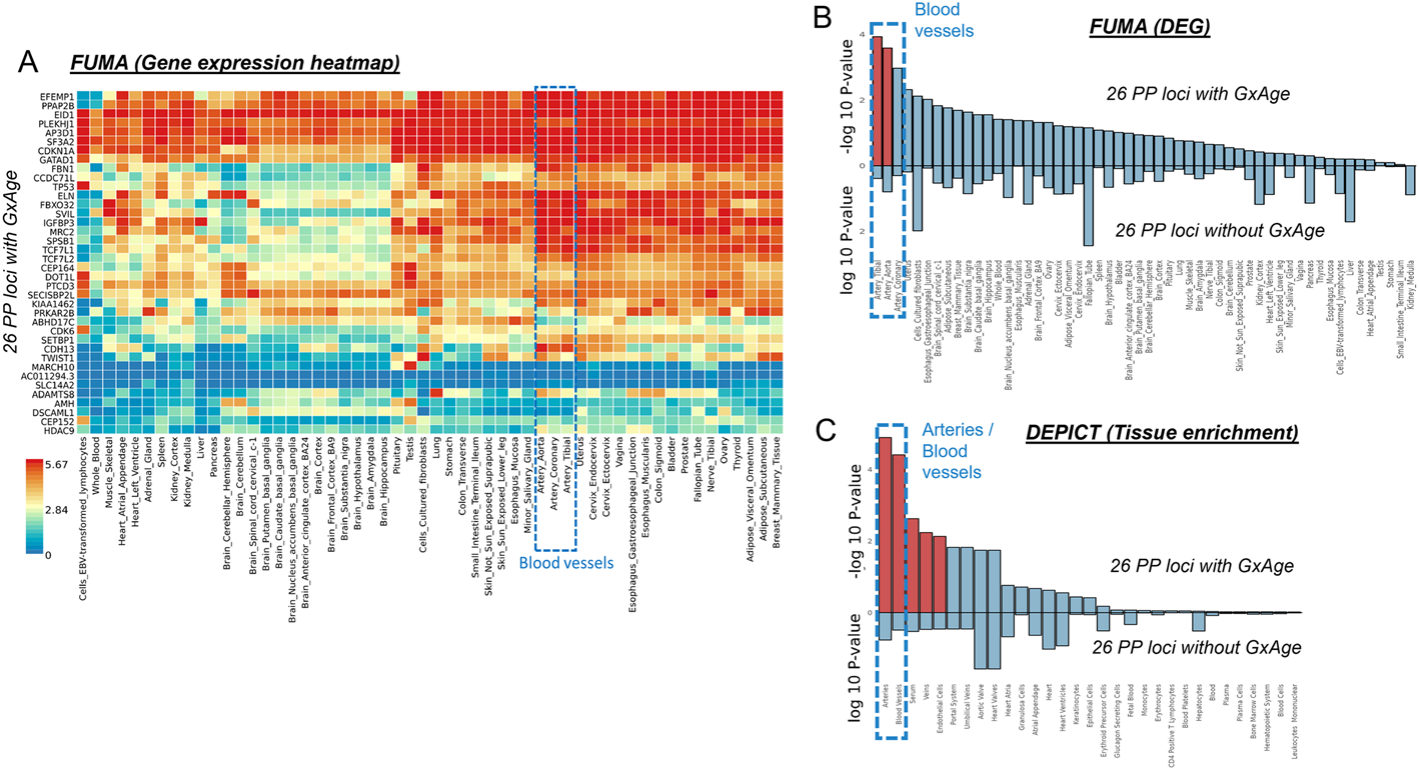
Tissue-specific enrichment of gene expression at pulse pressure loci. For the 26 genetic-by-age interaction loci (*P*_GxAge_ < 5 × 10^−8^ or significant in the 2-step approach, Additional file 1: Table S3), shown are **A** the clustered gene expression heatmap for 54 GTEx (v8) tissue types for the FUMA mapped genes (value shown is average expression per label, log2 transformed), **B** results from tissue-specific differentially expressed gene set enrichment analyses by FUMA (upper bars; significant enrichments highlighted in red, FDR < 5%), and **C** enrichment of gene expression analysis results by DEPICT for selected tissues and cell-types (upper bars; significant enrichments highlighted in red, FDR < 5%). For comparison, shown in **B** and **C**, respectively, are FUMA and DEPICT tissue-specific enrichment analysis results for 26 pulse pressure loci without genetic-by-age interaction (lower bars in **B**, **C**; i.e., *P*_Marginal_ < 5 × 10.^−8^ and *P*_GxAge_ > 0.48, Additional file 1: Table S5). Detailed FUMA results are shown in Additional file 1: Table S9. Results on all tissues by DEPICT are shown in Additional file 1: Table S10 and Additional file 2: Fig. S6

For pulse pressure, the genetic effects were intensified for 24 of the 26 variants (enrichment *P* = 1.0 × 10^−5^, Fig. 7B). The pulse pressure increasing alleles were enriched for nominal significant effects on increased pulse wave arterial stiffness index in UKB (enrichment *P* = 0.04, *N* = 118,469, GWAS summary statistics from the Neale lab, https://www.nealelab.is/uk-biobank, Additional file 1: Table S8). The observed genetic-by-age interaction effects for pulse pressure were not confounded by genetic-by-BMI interaction in sensitivity analyses (Additional file 1: Table S8). We conducted tissue-specific expression analyses with FUMA and observed significantly differentially expressed genes in tibial arteries and in aorta arteries (FDR < 5%, Fig. 8A and B, Additional file 1: Table S10). In terms of direction, the enrichment in arteries was particularly observed for upregulated gene expression (Additional file 1: Table S10, Additional file 2: Fig. S5). A confirmatory pattern was observed by enrichment analysis with DEPICT, which yielded significant enrichment of gene expression effects in 16 tissues and cell types including blood vessels, arteries, veins, and endothelial cells (tissue enrichment FDR < 5%, Fig. 8C, Additional file 2: Fig. S6, Additional file 1: Table S11). Strikingly, the enrichment in relevant tissues could not be found in complimentary enrichment analyses that were based on 26 pulse pressure loci without genetic-by-age interaction (i.e., associated but no genetic-by-age interaction; *P*_Marginal_ < 5 × 10^−8^ and *P*_GxAge_ > 0.48; Fig. 8B and C). This distinction was confirmed by direct variant-to-tissue mapping based on chromatin activity (FORGE2 [13]), which yielded a significant enrichment of blood vessel cells for the 26 genetic-by-age interaction pulse pressure loci (4 out of 14 blood vessel cells were mapped with nominal significance, binomial enrichment *P* = 0.004) but no enrichment for the 26 loci without genetic-by-age interaction (Additional file 1: Table S12). Together, these results underscore the biological relevance of age as intensifying modulator in pulse pressure genetics and biology.

In summary, we found an interesting distinction of traits based on their observed genetic-by-age interactions: those with intensified effects over age implicating aging processes of arterial stiffness (i.e., pulse pressure), those with attenuated effects over age due to an increasing impact of other environmental factors (i.e., BMI and weight), and those with relatively constant genetic effects over age (lipids).

## Discussion

In this work, we showed that genetic-by-age interaction analyses in cross-sectional data can successfully help identify genetic effects on longitudinal change. Using cross-sectional UKB data for > 370,000 individuals for eight complex traits, our GWAS of genetic-by-age interaction identified 44 significant variants across 36 independent loci. We observed a biological distinction of traits into those with plenty of identified genetic-by-age interactions, including obesity (7 genetic-by-age interaction loci for weight and BMI; 6 being attenuated with age) and pulse pressure (26 loci; 24 being intensified with age) versus those with very few or no genetic-by-age interactions (other blood pressure traits, lipid traits). Considering a broader spectrum of marginally associated trait variants confirmed these observed proportions and directions, with > 50% of pulse pressure variant effects being intensified, and ~ 15% of obesity variant effects being attenuated with age. Our results underscore the relevance of genetic-by-age interaction as being interpretable as genetic effects on trait change. They also highlight the biological implications of distinguishing between traits where their genetic make-up is, partly, age-dependent (i.e., genetic impact attenuated or intensified with age) or constant over age.

For obesity traits, the genetic effects on BMI or weight were attenuated with increased age towards zero for all variants (near *TMEM18*, *TFAP2B*, *EFR3B*, *SEC16B*, *FTO*, and additionally, *HMGN4* for weight) except for the variant near *APOE*. This pattern was consistent with previous work from the GIANT consortium that identified 11 loci with smaller genetic effect sizes on BMI among older compared to younger adults using age-stratified GWAS meta-analyses on cross-sectional data [14]. Four of the 11 loci by GIANT were validated by our analyses of BMI-change using independent longitudinal data from UKB (near *TMEM18*, *EFR3B*, *SEC16B*, and *FTO*). Reduced genetic effect sizes with increased age reflect a plausible biological pattern for BMI genetics that is prone to environmental effects [15, 16]: for example, effects of *FTO* are impacted by physical activity, alcohol consumption, or sleep duration [17], *TMEM18* by drinking habits and physical activity [18], and *SEC16B* by physical activity [19]. An attenuation of the genetic BMI effects by increased age is in-line with an accumulation of environmental impact on BMI that diminishes the genetic BMI effects. Interestingly, the identified variants map to genes that are likely acting on BMI through adipose tissue function, energy expenditure, or lipid metabolisms [20–24]. In contrast, variants known to exhibit their effects on BMI in the brain through appetite regulation, such as variants in another well-studied BMI locus near *MC4R* [25, 26], showed no significant genetic-by-age interaction, which may suggest that they are less prone to environmental impacts.

For pulse pressure, we identified a striking enrichment of 26 loci with significant genetic-by-age interactions and a clear picture in terms of the interaction effect directions. Only the locus near *PIK3CG* was mentioned before for potential age-dependent effect on pulse pressure, however only with nominal significance despite multiple testing [27]. At 24 of the 26 variants, the genetic effects on pulse pressure were clearly increased with age; two were undecided. The 26 loci were also enriched for upregulated gene expression effects in arteries and blood vessel tissues consistently by FUMA and DEPICT analyses, which supports them to pinpoint relevant pulse pressure biology. Gene prioritization analyses by DEPICT and FUMA yielded 11 genes that were prioritized by both methods: *CDH13*, *CDKN1A*, *DOT1L*, *EFEMP1*, *FBN1*, *FBXO32*, *IGFBP3*, *KIAA1462*, *MRC2*, *SPSB1*, *TCF7L1*. These include interesting candidates such as *IGFBP3* (insulin-like growth factor binding protein-3) that has previously been reported for its association with ankle brachial index in a cohort of elderly [28] or *CDH13* (cadherin 13), which is a known regulator of vascular wall remodeling [29], and *FBN1* (fibrilin-1) that is known for its impact on arterial stiffness [30]. The results support pulse pressure as aging index for arterial stiffness [31] and highlight the relevance of the genetic-by-age interaction loci for biological mechanism that affect accelerated arterial stiffness, which is not existent at younger age but increases with ascending age.

We demonstrated that genetic-by-age interaction can be equivalent to the genetic effect on trait change under certain assumptions. These assumptions are random baseline timepoint, linear effect of age on trait, negligible calendar time and birth cohort effect, and constant effects of other covariates over age. In case of relevant non-constant covariate effects like sex-by-age interaction on the trait, stratified analyses by sex can be conducted. In case of a non-linear effect of age on trait, trait transformation or adding age^2^ to the model can improve the linear model fit. The assumptions can be compromised by birthyear effects in the cross-sectional data as well as age-dependent selection or survival. While birthyear effects can be adjusted for, in theory, it is almost impossible to dissect birthyear effects from age effects in practice when birthyear and age are highly correlated. This is the case in UKB due to the short time span of recruiting. We show the similarity of effect sizes for genetic-by-age effects and variant’s effect on trait change also empirically—for the variants that were validated in longitudinal data. This supports the “2-stage GxAgeChange” approach to yield robust evidence for genetic variant effects on trait change. An indicator for birthyear, survival, or selection effects on a genetic variant is the genetic variant’s effect on age. We found none of the identified 44 variants associated with age, except for the *APOE* variant which is known for association with Alzheimer’s and early mortality [32–34]. The *APOE* variant’s association with age may thus be explained by age-dependent selection or survival rather than a birthyear effect. We recommend the “2-stage GxAgeChange” approach together with a test of identified genetic variants for association with age.

We showed that the power to identify genetic effects on change was increased by pre-screening on genetic-by-age interaction in cross-sectional data in this UKB dataset, where the sample size of the cross-sectional data substantially exceeds the longitudinal data (internal data pre-screening). The “2-stage GxAgeChange” can also be applied using external data for pre-screening, like genetic-by-age interaction analyses via meta-analyses followed by testing for trait change in an independent longitudinal study.

Our relatively simple 2-stage approach of pre-screening for genetic-by-age interaction in cross-sectional data and testing of genetic association with trait change in longitudinal data successfully identified longitudinal change effects in this UKB dataset. Among the 44 genetic-by-age interactions observed on the cross-sectional data, 19 were validated for directionally consistent and nominal significant effects on annual change of the respective trait in up to 50,000 independent individuals with longitudinal data from UKB. These included seven associations for trait change that were significant at a Bonferroni-corrected significance level: two for BMI-change (near *APOE*, *TMEM18*), three for weight-change (near *APOE*, *TMEM18*, and *TFAP2B*), and two for pulse pressure change (*FBN1* and *IGFBP3*). For obesity, *TMEM18* and *TFAP2B* were identified here for the first time as loci for BMI or weight change. They were missed by a longitudinal GWAS using electronical health records (EHRs) from UKB despite larger longitudinal sample size of ~ 170,000 and inclusion of multiple timepoints per person [4]. For pulse pressure, we could not identify a previous longitudinal GWAS, so that our identified loci are the first reports of genetic variants associated with longitudinal pulse pressure change.

Some limitations need to be acknowledged. First, we assumed a linear age effect on the trait and might have some non-linear age component in the trait unaccounted for; also, we searched for genetic variants associated with linear trait change over age and thus might have missed genetic variants with non-linear change. Second, we have only considered assessment center data from UKB with a limited number of individuals with two longitudinal timepoints per individual (15 K to 50 K, trait-dependent). Third, we analyzed annual change of a trait based on two timepoints per individual using standard linear regression; linear mixed models (LMMs) including random effects would allow for analyzing multiple longitudinal timepoints per individual. In UKB, multiple trait timepoints per person would generally be accessible from EHRs [35]. While incorporating LMMs and data on multiple timepoints will improve power of the longitudinal data analysis itself, their implementation in biobank-scale GWAS is computationally more intense and the data preparation more challenging [35]. Another limitation of our study is the focus on the European population and to metabolic traits. Yet, the 2-stage approach is readily applicable to non-European populations and to other traits [36]. Finally, we have used chronological age that may not necessarily reflect true biological age [37]. Future work may incorporate novel approaches to estimate true biological age based on OMICs data [38] and use this as covariate and as interaction variable to improve the identification and characterization of age-dependent genetics of complex traits. Biological aging can differ from chronological aging at the molecular level. Age clocks based on telomere length and age-dependent DNA methylation CpG sites reflect this process [39]. Also, the aging immune system, the balance of antibody-producing cells and T cells is strongly age-dependent and has an impact on the ability to respond to several infections [40]. Thus, any observed genetic-by-age interaction could potentially be explained by genetic-by-CpG interactions or be linked to mechanisms that are related to the age-dependent immune system. Our results emphasize the importance to account for age-dependency in several aspects of GWAS, such as fine mapping or heritability estimation, and may open the route to future methods developments.

Our results suggest that genetic-by-age interaction might be underacknowledged regarding their potential to understand biology and aging across traits.

## Conclusions

In summary, we demonstrate that genetic-by-age interaction testing in cross-sectional data can help identify genetic association with trait change in longitudinal data. Our work highlights obesity and pulse pressure as traits that have a substantial component of genetic-by-age interaction in cross-sectional data. These can highlight differential biological processes that are age-related versus constant over age. In contrast, lipid traits showed little evidence for genetic-by-age interactions. This might also indicate that we can expect more from longitudinal GWAS for traits related to obesity and pulse pressure rather than for lipids. The observation that genetic effect sizes on pulse pressure become larger by older age suggests that the identified loci predispose to accelerated aging processes. This highlights the relevance of considering age as potential modulator of genetic effects to help understand mechanisms of aging.

## Methods

### UK Biobank

The UKB included approximately 500,000 individuals from the UK aged 40–69 years. The samples were genotyped based on the Affymetrix UKB Axiom Array and then imputed to the Haplotype Reference Consortium and the UK10K haplotype resource [41]. We restricted our analysis sample to individuals of European population using the population definitions generated by the PAN-UKB project (https://pan.ukbb.broadinstitute.org/).

### Phenotype definitions

We conducted genome-wide association and genetic-by-age interaction analyses for eight traits. Five of the traits were available at four UKB visits: weight, BMI, diastolic blood pressure (DBP), systolic blood pressure (SBP), and pulse pressure (PP). The three other traits were only available from the baseline and the first repeat visit: high-density lipoprotein cholesterol (HDL-C), low-density lipoprotein cholesterol (LDL-C), and triglycerides. For weight and BMI, we used the UKB variables 21,002 and 21,001 directly, respectively. For both DBP and SBP, two measurements were available at each visit (UKB variables 4079 and 4080). We calculated the mean of the two variables, respectively, and then added 10 mmHg to the mean DBP and 15 to the mean SBP value of an individual if the person took anti-hypertensive medication indicated by self-report (“Blood pressure medication” in UKB variable 6177) or by medication Anatomical Therapeutic Chemical (ATC) code (“C02,” “C03,” “C07,” “C08,” or “C09” in UKB variable 20,003). PP was calculated as the difference between the medication adjusted SBP and DBP values (PP = SBP − DBP). The three traits DBP, SBP, and PP were winsorized at ± 6 standard deviations. For the lipid traits of HDL-C, LDL-C, and triglycerides, we used the UKB variables 30,760, 30,780, and 30,870, respectively, for the traits measured in mmol/l. We multiplied HDL-C and LDL-C by 38.67 and triglycerides by 88.57 to obtain values in mg/dl. We applied a natural log-transformation to the derived HDL-C and triglyceride values to obtain symmetric outcomes. The LDL-C values were not subject to log transformation but were divided by 0.7 if the person took cholesterol lowering medication indicated by self-report (“Cholesterol lowering medication” in UKB variable 6177) or by medication ATC code (“C10” in UKB variable 20,003). The natural log-transformed HDL-C and triglyceride values and the medication-adjusted LDL-C values were winsorized at ± 6 standard deviations (SD).

### Longitudinal annual change

We defined longitudinal annual outcome change for the traits as follows: For traits with more than two measurements available (BMI and blood pressure traits), we used the first available follow-up visit value (e.g., if data is available from “first repeat,” then we use “first repeat”; if not we try to use the “imaging” visit and if not, then finally the “repeat imaging” visit was used) and subtracted the baseline value to obtain the absolute difference between the follow-up and the baseline visit. We further divided by the time between visits to obtain annual change of the outcome. For lipid traits, we always use the repeat and the baseline visit to obtain annual change.

### Three approaches to search for annual change effects

We consider three approaches to identify annual change effects: (i) The genome-wide screen for genetic-by-age interaction in cross-sectional data. This approach consists of a genetic-by-age interaction test genome-wide (judging significant interaction by *P*_GxAge_ < 5 × 10^−8^) and a genetic-by-age interaction testing focused to variants with genome-wide significant marginal effects (*P* < 5 × 10^−8^; then *P*_GxAge_ < *M*_eff_, with *M*_eff_ being the number of effective tests among marginally associated variants estimated from a principal component analysis) [42]. The significant genetic-by-age interaction variants are clumped to derive genetic-by-age interaction loci (*d* > 500 kb; *r*^2^ < 0.01) and index variants are selected (i.e., variant with smallest genetic-by-age interaction *P* value among variants at a locus). (ii) The genome-wide screen for genetic-by-age interaction in cross-sectional data (same as (i)) with additional validation association testing for annual trait change effects in independent longitudinal data (using a Bonferroni-correction based on the number of identified genetic-by-age index variants). (iii) The genome-wide screen for annual change effects in longitudinal data. Like the first approach, this approach consists of an annual change association test genome-wide (judging significant change effects by *P*_Change_ < 5 × 10^−8^) and an annual change association test focused to variants with genome-wide significant marginal effects (*P*_Change_ < 0.05/*M*_eff_).

### Power computations

We generally assumed unrelated individuals, an additive genotype model with a fixed allele frequency of 30%, and UKB-based phenotype and sample size configurations (Table 1, Additional file 1: Table S1). Details and analytical power formulae for three “1-stage GxAge,” the “1-stage Change,” and the “2-stage GxAgeChange” approaches are shown in Additional file 2: Note S1. To derive power for the genome-wide GxAge search (PWR_GxAge,gws_), we calculated power to identify an interaction effect at alpha_GxAge_ = 5 × 10^−8^. To derive power of the 2-step GxAge search (PWR_GxAge,2-step_), we first calculated power to find a marginal effect at alpha_G_ = 5 × 10^−8^ and multiplied this by the power to identify an interaction effect at alpha_GxAge_ = 0.05/1000 (assuming 1000 independent interaction tests among marginally associated variants). Power of the “1-stage GxAge” approach was then calculated by a combination of the two approaches: PWR_GxAge_ = PWR_GxAge,gws_ + PWR_GxAge,2-step_ − PWR_GxAge,gws_ * PWR_GxAge,2-step_. Power to identify annual change effects in 1-stage was calculated similarly based on a combination of a genome-wide search, alpha_Change_ = 5 × 10^−8^, and a 2-step search, alpha_Change_ = 0.05/1000 (again assuming 1000 independent tests among marginally associated variants). For the “2-stage GxAgeChange” approach with validation for annual change effects, power of the GxAge approach (PWR_GxAge_) was multiplied with the power to identify annual change effects at alpha_Change_ = 0.05/10 (assuming 10 independent GxAge interactions).

### Genome-wide association analyses

We used regenie [43] to conduct our genome-wide association and genetic-by-age interaction analyses. We assumed an additive genotype model and employed a linear mixed model to account for population substructure and further adjusted the regression analyses for sex, age, age × sex, and 20 genetic principal components obtained from the PAN-UKB project (https://pan.ukbb.broadinstitute.org/). We excluded any variants that were rare (MAF < 0.01%) and restricted our analyses to variants with high imputation quality (Info ≥ 0.8). We applied a genomic control correction to the genome-wide association and genetic-by-age interaction results (using GC lambdas that were estimated based on null variants > 5 Mb distant from genome-wide significant marginally associated variants) [44]. Within regenie, we applied the “–interaction agec –no-contl” parameter in order to fit the genetic-by-age interaction model and to additionally output marginal (unconditioned) genetic effect estimates. The marginal effect sizes were then utilized in the 2-step approaches (pre-filtering on marginal effects). For the annual trait change GWAS in the longitudinal data, we employed a regular regenie analysis without interaction parameter.

### Variant selection and locus definition

To derive non-overlapping loci and their index variants, we first clumped the significant variants into independent regions based on a base position threshold of 500 kb (i.e., distance between significant regions is always greater than 500 kb). Then, within each region, we further clumped the significant variants into loci based on a *r*^2^ threshold of 0.01: Starting with the most significant variant of the region as the index variant of the first LD bin, we added all correlated variants (*r*^2^ ≥ 0.01) to this first locus. We removed all variants of the first locus and restarted the clumping taking the most significant variant among remaining variants as the index variant of the second locus. We stopped when there were no more variants left within the region and continued to clump loci in the next region. We utilized a reference file of 20,000 unrelated individuals of the UKB to obtain *r*^2^.

### Sensitivity analyses to evaluate birth cohort, selection, and survival effects

A genetic-by-age interaction (GxAge) effect on the trait *Y* can be confounded by a covariate *C* that is (i) correlated with GxAge and associated with *Y* (explored by adjusting for *C*), (ii) correlated with age and exerting a genetic-by-covariate interaction (GxC) with the trait (explored by adjusting for GxC), or (iii) correlated with *G* and the covariate-by-age interaction (CxAge) being associated with the trait (explored by adjusting for CxAge) [7]. Birthyear can be such a confounder (birth cohort effect). In theory, this can be explored by sensitivity analyses adjusting for birthyear, GxBirthyear, and AgexBirthyear. Yet, in practice, age and birthyear are often highly correlated in cohort studies leading to collinearity and thus inconclusive results for such sensitivity analyses. We explored this correlation and sensitivity analysis adjusting for birthyear:

$$Y={\beta }_{0}+{\beta }_{G}G+{\beta }_{Age}AGE+{\beta }_{GxAge}G\cdot AGE+{\beta }_{BY}BY+{\beta }_{C}C+\varepsilon$$

When birthyear and *AGE* are highly correlated and birthyear affects *G*, the genetic variant *G* will be associated with *AGE*: this can be tested in practice by fitting a linear regression model with *AGE* as outcome,

$$AGE={\delta }_{0}+{\delta }_{G}G+{\delta }_{C}C+\epsilon$$

An association of *G* with age is not only possible due to a birthyear effect, but can also result from survival or selection effects. We tested each variant identified with GxAGE for association with *AGE* with this model.

### Gene prioritization and tissue-specific enrichment

We conducted gene prioritization and tissue-specific enrichment analyses at the genetic-by-age interaction loci using FUMA [10] and DEPICT [9] as well as direct variant-to-tissue mapping using FORGE2 [13]. For FUMA, significant genetic-by-age interaction variants were uploaded to the SNP2GENE portal whereas the identified index variants and genomic regions were used as predefined lead variants and regions (http://fuma.ctglab.nl/). The option to identify additional index variants was switched off to ensure gene mapping at the identified variants. Gene mapping was based on position, expression, and 3D chromatin interaction (both restricted to relevant tissues). Results from the SNP2GENE mapping were transferred to GENE2FUNC mapping functionality to obtain tissue specific expression analysis results and expression heatmaps. DEPICT analysis were conducted based on variants located at the identified genetic-by-age interaction loci with a relaxed *P* value threshold of *P*_GxAge_ < 1 × 10^−5^ on the virtual analysis platform Complex Traits Genetics Virtual Lab (CTG-VL, https://vl.genoma.io/) [45]. FORGE2 mapping based on chromatin activity was conducted based on ENCODE annotations for the genetic-by-age interaction variants using the FORGE2 online tool (https://forge2.altiusinstitute.org/). DEPICT, FUMA, and FORGE2 analyses were only informative for pulse pressure and could not be executed for the other traits due to the relatively small number of genetic-by-age interaction loci identified. For comparison reasons, FUMA, DEPICT, and FORGE2 analyses were repeated for genome-wide significant pulse pressure loci without genetic-by-age interaction (*P*_Marginal_ < 5 × 10^−8^; but no interaction with age).

## Supplementary Information

Additional file 1: Table S1. Study descriptives. Table S2. Controlled power. Table S3. Descriptive summary of GWAS. Table S4. Gene-age interaction loci. Table S5. Gene prioritization by DEPICT and FUMA at 44 genetic-by-age interaction loci. Table S6. Marginal effect region lead variants. Table S7. Sensitivity analyses for cohort and selection/survival effects. Table S8. Pulse wave arterial stiffness index in UKB. Table S9. Sensitivity analysis adjusting for GxBMI. Table S10. Tissue enrichment analyses by FUMA for genetic-by-age interaction loci for pulse pressure. Table S11. Tissue enrichment analyses by DEPICT for genetic-by-age interaction loci for pulse pressure. Table S12. FORGE2 variant-to-tissue mapping.

Additional file 2: Note S1. Mathematical equivalence of gene-age interaction and genetic annual outcome change effects. Note S2. Analytical power computation. Note S3. Equivalence of power between a genetic-by-age interaction and an annual change association test. Fig. S1. Power to identify genetic-by-age interaction and longitudinal change effects. Fig. S2. Miami plots comparing genetic-by-age interaction with marginal effect associations. Fig. S3. Heatmap of genetic-by-interaction effect sizes. Fig. S4. Comparison of gene-age interaction and marginal effect sizes. Fig. S5. FUMA differentially up- and downregulated expressed genes for pulse pressure loci with genetic-by-age interaction. Fig. S6. DEPICT tissue and cell-type specific enrichment for pulse pressure.

Additional file 3. Review history.

## Data Availability

The analysis code for the application of the three approaches is available under a Creative Commons Attribution 4.0 International License from github (https://github.com/genepi-regensburg/gxage) [46] and Zenodo (https://doi.org/10.5281/zenodo.14140293) [47]. Genome-wide summary statistics are available from www.genepi-regensburg.de/gwas-summary-statistics [48] and from Zenodo https://doi.org/10.5281/zenodo.14141226 [49]. The raw datasets supporting the conclusions of this article can be applied for from UK Biobank (https://www.ukbiobank.ac.uk/).
