# Supplementary material for: Genetic-by-age interaction analyses on complex traits in UK Biobank and their potential to identify effects on longitudinal trait change
Source: Genome Biol. 2024 Nov 28;25:300. doi: 10.1186/s13059-024-03439-9 (PMC11606088; doi:10.1186/s13059-024-03439-9)
Supplement: Supplementary file 2 — Additional file 2: Note S1. Mathematical equivalence of gene-age interaction and genetic annual outcome change effects. Note S2. Analytical power computation. Note S3. Equivalence of power between a genetic-by-age interaction and an annual change association test. Fig. S1. Power to identify genetic-by-age interaction and longitudinal change effects. Fig. S2. Miami plots comparing genetic-by-age interaction with marginal effect associations. Fig. S3. Heatmap of genetic-by-interaction effect sizes. Fig. S4. Comparison of gene-age interaction and marginal effect sizes. Fig. S5. FUMA differentially up- and downregulated expressed genes for pulse pressure loci with genetic-by-age interaction. Fig. S6. DEPICT tissue and cell-type specific enrichment for pulse pressure. [file 13059_2024_3439_MOESM2_ESM.docx]

Additional file 1. Supplementary material

**Note S1**. Mathematical equivalence of gene-age interaction and genetic annual outcome change effects.

**Note S2**. Analytical power compuation.

**Note S3**. Equivalence of power between a genetic-by-age interaction and an annual change association test.

**Fig S1**. Power to identify genetic-by-age interaction and longitudinal change effects.

**Fig S2**. Miami plots comparing genetic-by-age interaction with marginal effect associations.

**Fig S3**. Heatmap of genetic-by-interaction effect sizes.

**Fig S4**. Comparison of gene-age interaction and marginal effect sizes.

**Fig S5**. FUMA differentially up- and down-regulated expressed genes for pulse pressure loci with genetic-by-age interaction.

**Fig S6**. DEPICT tissue and cell-type specific enrichment at pulse pressure loci.

**References.**

## **Note S1. Mathematical equivalence of gene-age interaction and genetic annual outcome** change effects.

We here demonstrate under which conditions the genetic-by-age interaction effect, $\hat{\beta}_{GxAge}$, is equivalent to the genetic effect on annual trait change, $\hat{\gamma}_{G}$. For longitudinal data, we assume two time points (*t_1_* and *t_2_*) and denote the corresponding individual’s trait values as *Y_1_* and *Y_2_* and the corresponding individual’s age as *AGE_1_* and *AGE_2_*, respectively. For cross-sectional data, we denote the individual’s trait value with *Y* and the corresponding age with *AGE*.

For longitudinal data, a genetic variant, *G*, can be evaluated for association with annual trait change by the linear regression model:

$\frac{Y_{2}-Y_{1}}{t_{2}-t_{1}}=\gamma_{0}+\gamma_{G}G+ \gamma_{Age}{AGE}_{1}+\gamma_{C}C+\varepsilon,$ (1)

where the left-hand side – the annual trait change – is the difference in $Y$ between time points *t_2_* and *t_1_*, divided by the time passed between the two time points. An example for *C* is sex or principal components.

We make the following assumptions: (i) We assume that the 1^st^ timepoint is random and does not mark an intervention, which typically is the case in observational data (“random baseline assumption”). (ii) We also assume that the change in *Y* over time primarily reflects the aging of individuals. In addition, there might be a calendar time effect, i.e., *Y* might change by calendar time independent of the individual’s age. Such effects may arise due to substantial change in the environment. When the two time points of the longitudinal data do not span several decades, such calendar time effects are less likely. We thus assume here no or negligible calendar time effect on *Y* (“no calendar time effect assumption”). (iii) We further assume that *Y* changes over age linearly (“linearity assumption”). This implies that age at baseline has no effect on annual change of Y, $\gamma_{Age}=0$. (iv) We assume that the effect of *G* on the change of *Y* over time reflects the effect of *G* on the change of *Y* over age, i.e., that the effect of *G* on *Y* is independent of calendar time (“no calendar time effect assumption”). (v) Finally, we assume that the effect of covariate *C* on *Y* is independent of calendar time (e.g., no sex-by-calendar time interaction on Y; “no calendar time effect assumption”) and age (e.g. no sex-by-age interaction effect on Y; “constant covariate effect assumption”). This implies that *C* has no effect on annual change of Y, $\gamma_{C}=0$ (e.g., no sex effect on the annual change of *Y*). Under these assumptions, (1) simplifies to

$\frac{Y_{2}-Y_{1}}{{AGE}_{2}-{AGE}_{1}}=\gamma_{0}+\gamma_{G}G+\varepsilon$ (2)

Noteworthy, a birthyear effect on Y is eliminated here by the subtraction. We can rewrite (2) as

$Y_{2}-Y_{1}=\gamma_{0}\left( {AGE}_{2}-{AGE}_{1} \right)+\gamma_{G}G*\left( {AGE}_{2}-{AGE}_{1} \right)+\varepsilon\left( {AGE}_{2}-{AGE}_{1} \right)$ (3)

For the cross-sectional analyses of genetic-by-age interactions, we conduct linear regression with the trait *Y* as outcome (age centered at mean age, here 56 years):

$Y=\beta_{0}+\beta_{Age}AGE+\beta_{G}G+\beta_{GxAge}G*AGE+\beta_{C}C+\varepsilon$ (4)

This model assumes (i) that the effect of age on *Y* is linear (“linearity assumption”), (ii) and that the effect of C on Y is independent of age (“constant covariate effect assumption”). We also assume (iii) that there are no birth cohort effects: levels of *Y* depend on age but not on birthyear, effect of *G* on *Y* is independent of birthyear, effect of *C* on *Y* is independent of birthyear (“no birth cohort effect assumptions”). Let us consider applying model (4) for cross-sectional data taken at two timepoints,

$Y_{1}=\beta_{0}+\beta_{Age1}{AGE}_{1}+\beta_{G1}G+\beta_{GxAge1}G*{AGE}_{1}+\beta_{C1}C_{1}+\varepsilon_{1}$ (5)

and

$Y_{2}=\beta_{0}+\beta_{Age2}{AGE}_{2}+\beta_{G2}G+\beta_{GxAge2}G*{AGE}_{2}+\beta_{C2}C_{2}+\varepsilon_{2}$ (6).

Note that *G* is the same at both time points. We assume $\beta_{Age1}=\beta_{Age2}$, $\beta_{G1}=\beta_{G2}$, $\beta_{GxAge1}=\beta_{GxAge2}$, and $\beta_{C1}=\beta_{C2}$. i.e., that there is no calendar time effect for the age, G, GxAge and C effect on Y. Then, the difference of *Y_2_* and *Y_1_* can be written as

$Y_{2}-Y_{1}=\beta_{Age}\left( {AGE}_{2}-{AGE}_{1} \right)+\beta_{GxAge}G*\left( {AGE}_{2}-{AGE}_{1} \right)+\left( \varepsilon_{2}-\varepsilon_{1} \right)$ (7)

Assuming i.i.d. random noise error terms $\varepsilon$ throughout, comparing (3) and (7) shows the equivalence between the genetic effect on annual *Y* change, $\gamma_{G}$, with the genetic-by-age interaction effect on *Y*, $\beta_{GxAge}$, i.e., $\gamma_{G}$≅$\beta_{GxAge}$.

## **Note S2. Analytical power computation**.

We derived analytical power formulas for the three approaches, including the “1-stage GxAge” approach, the “1-stage Change” approach and the “2-stage GxAge🡪Change” approach. The three approaches involve association Wald tests of genetic-by-age interaction effects, marginal effects or genetic annual change effects. Power of the Wald test is generally given by

| ${Pwr}_{t}\left( \alpha,R^{2},n \right)=t_{n-2}\left( -t_{n-2,1-\frac{\alpha}{2}}-\sqrt{\frac{nR^{2}}{1-R^{2}}} \right)+t_{n-2}\left( -t_{n-2,1-\frac{\alpha}{2}}+\sqrt{\frac{nR^{2}}{1-R^{2}}} \right)$ (8) |  |
| --- | --- |

where $t_{n-2}$ denotes the cumulative distribution function of a t distribution with $n-2$ df and $t_{n-2,q}$ denotes the q-th quantile of $\Phi$, $\alpha$ is the alpha-level, n is the sample size and $R^{2}$ is the explained variance of the predictor [2]. For marginal and annual change genetic effects that are both estimated in a linear regression framework (without interaction), the explained variance of the genetic effect can be calculated by $R_{G}^{2}(\hat{\beta}_{G}, s_{G}^{2},s_{Y}^{2})=\hat{\beta}_{G}\frac{s_{G}^{2}}{s_{Y}^{2}}$, where $\hat{\beta}_{G}$ is the genetic effect, $s_{Y}^{2}$ is the variance of the outcome , $s_{G}^{2}$ is the variance of the genotype (can be calculated by $s_{G}^{2}=2*AF*\left( 1-AF \right)$, with *AF* being the allele frequency of a genetic variant). For genetic-by-age interaction effects that are estimated in a linear regression framework with genetic-by-age interaction term, the explained variance by the genetic-by-age interaction can be calculated by $R_{GxAge}^{2}(\hat{\beta}_{GxAge}, s_{G}^{2},s_{Y}^{2},s_{Age}^{2})=\hat{\beta}_{GxAge}\frac{s_{Age}^{2}s_{G}^{2}}{s_{Y}^{2}}$, where $\hat{\beta}_{GxAge}$ is the genetic gene-age interaction effect and $s_{Age}^{2}$ is the variance of the interacting factor age [3]. Based on these assumptions, power of the three approaches can be calculated as follows:

1. “1-stage GxAge” approach: This approach combines a genome-wide genetic-by-age interaction test ($\alpha_{GxAge}$=$5x{10}^{-8}$) and a 2-step genetic-by-age interaction test (involves testing for marginal association at $\alpha_{G}$=$5x{10}^{-8}$ in step 1; and testing filtered variants for genetic-by-age interaction at $\alpha_{GxAge}=0.05/M_{eff}$, with M_eff_ being the effective number of independent tests). Thus, power can be expressed as

${Pwr}_{1stageGxAge}={Pwr}_{t}\left( 5x{10}^{-8},R_{GxAge}^{2},n_{nolong} \right)+{Pwr}_{t}\left( 5x{10}^{-8},R_{G}^{2},n_{nolong} \right)*{Pwr}_{t}\left( 0.05/M_{eff},R_{G}^{2},n_{nolong} \right)$-${Pwr}_{t}\left( 5x{10}^{-8},R_{GxAge}^{2},n_{nolong} \right)$*${Pwr}_{t}\left( 5x{10}^{-8},R_{G}^{2},n_{nolong} \right)*{Pwt}_{t}\left( 0.05/M_{eff},R_{G}^{2},n_{nolong} \right)$,

where $n_{nolong}$ is the sample size (after excluding individuals with longitudinal data), $R_{GxAge}^{2}=R_{GxAge}^{2}(\hat{\beta}_{GxAge}, s_{G}^{2},s_{Y}^{2},s_{Age}^{2})$, i.e., the explained variance by the genetic-by-age interaction and $R_{G}^{2}=R_{G}^{2}(\hat{\beta}_{G}, s_{G}^{2},s_{Y}^{2})$, i.e., the explained variance by the marginal genetic effect.

1. “1-stage Change” approach: This approach combines a genome-wide genetic annual change association test ($\alpha_{Change}$=$5x{10}^{-8}$) and a 2-step test (involves testing for marginal association at $\alpha_{G}$=$5x{10}^{-8}$ in step 1; and testing filtered variants for annual change at $\alpha_{Change}=0.05/M_{eff}$, with M_eff_ being the effective number of independent tests). Thus, power can be expressed as

${Pwr}_{1stageChange}={Pwr}_{t}\left( 5x{10}^{-8},R_{Change}^{2},n_{long} \right)+{Pwr}_{t}\left( 5x{10}^{-8},R_{G}^{2},n_{nolong} \right)*{Pwr}_{t}\left( 0.05/M_{eff},R_{Change}^{2},n_{long} \right)$-${Pwr}_{t}\left( 5x{10}^{-8},R_{Change}^{2},n_{long} \right)$*${Pwr}_{t}\left( 5x{10}^{-8},R_{G}^{2},n_{nolong} \right)*{Pwr}_{t}\left( 0.05/M_{eff},R_{Change}^{2},n_{long} \right)$, where $n_{long}$ is the longitudinal sample size, $R_{Change}^{2}=R_{G}^{2}(\hat{\beta}_{Change}, s_{G}^{2},s_{Ychange}^{2})$, i.e., the explained variance (of annual Y-change) by the genetic annual change effect $\hat{\beta}_{Change}$.

1. “2-stage GxAge🡪Change” approach: This 2-stage approach combines a first stage that is identical to the “1-stage GxAge” approach with a second stage that is to follow up identified genetic-by-age interaction loci in longitudinal data for their annual change effects. Power of this approach can be expressed as ${Pwr}_{2stageGxAge\to Change}={Pwr}_{1stageGxAge}*{Pwr}_{t}\left( 0.05/M_{GxAge},R_{Change}^{2},n_{long} \right)$, where $M_{GxAge}$ is the number of identified variants from stage 1.

## **Note S3.** Comparison of power between a genetic-by-age interaction and an annual change association test.

In the following, we compare the power of a genetic-by-age interaction (GxAge) test with the power of an annual change association test in a controlled setting. We assume the same number of individuals in the cross-sectional and in the longitudinal data, N=N_GxAge_=N_Change_=200K and alpha=alpha_GxAge_=alpha_Change_=5x10^-8^. By formula (8), power of the GxAge test is given as ${{Pwr}_{GxAge}=Pwr}_{t}\left( 5x{10}^{-8},R_{GxAge}^{2},200K \right)$ and power of the change test is given by ${{Pwr}_{Change}=Pwr}_{t}\left( 5x{10}^{-8},R_{Change}^{2},200K \right)$. Thus, power of the two tests is equivalent for $R_{GxAge}^{2}=R_{Change}^{2}$, and thus $\hat{\beta}_{GxAge}\frac{s_{Age}^{2}s_{G}^{2}}{s_{Y}^{2}}=\hat{\gamma}_{G}\frac{s_{G}^{2}}{s_{Change}^{2}}$ with $s_{Age}^{2}$ and $s_{Y}^{2}$ being the variance of *Age* and *Y* in the cross-sectional data, and $s_{Change}^{2}$ being the variance of the annual change of *Y* in the longitudinal data, and $s_{G}^{2}$ being the variance of *G* (assumed to be the same in both datasets). Further assuming $\hat{\beta}_{GxAge}=\hat{\gamma}_{G}$ (**Note S1**), this simplifies to

| $\frac{s_{Age}^{2}}{s_{Y}^{2}}=\frac{1}{s_{Change}^{2}}$ | (9) |
| --- | --- |

In the UKB longitudinal data, the follow-up time is nearly constant for all individuals (~4 or 7 years, dependent on trait) and the variance of *Y* at timepoint *t_1_* is similar to the variance of *Y* at timepoint *t_2_* for all eight traits (**Table 1, Table S1**). When assuming constant follow-up time and thus constant difference between *age_2_* and *age_1_* for all individuals, *age_diff_,* and assuming equivalent variance of *Y* at the two timepoints, $Var(Y_{1})=Var(Y_{2})$, then $s_{Change}^{2}$ can be expressed as follows:

$$s_{Change}^{2}=Var\left( \frac{Y_{2}-Y_{1}}{{age}_{2}-{age}_{1}} \right)=$$

$$=\frac{1}{{age}_{diff}^{2}}Var\left( Y_{2}-Y_{1} \right)=$$

$$=\frac{1}{{age}_{diff}^{2}}\left[ Var\left( Y_{2} \right)+Var\left( Y_{1} \right)-2r_{Y_{1},Y_{2}}\sqrt{Var\left( Y_{2} \right)}\sqrt{Var\left( Y_{1} \right)} \right]=$$

$=\frac{2*Var\left( Y_{1} \right)}{{age}_{diff}^{2}}\left[ 1-r_{Y_{1},Y_{2}} \right]$.

When assuming the variance of Y in the cross-sectional data ($s_{Y}^{2}=Var(Y)$) is the same as the variance of *Y* in the longitudinal data, i.e. $Var(Y)=Var(Y_{1})$, (9) can thus be written as

| $2*s_{Age}^{2}=\frac{{age}_{diff}^{2}}{1-r_{Y_{1},Y_{2}}}$ | (10) |
| --- | --- |

This implies: (i) the larger $s_{Age}^{2}$, the larger the power of the GxAge test. (ii) The longer the follow-up time (i.e. larger *age_diff_*) and the higher the correlation between *Y_1_* and *Y_2_* (i.e., lower technical measurement error or lower intra-individual variability), the larger the power of the change test.

## **Fig S1.** Power to identify genetic-by-age interaction and longitudinal change effects**.**

Shown are power curves for genetic-by-age interaction and annual change effects on A: Weight, B: HDL-C, C: TG, D: DBP and E: SBP. Power is shown for the three approaches: the “1-stage GxAge” approach in cross-sectional data (blue), the “2-stage GxAge🡪Change” approach that includes additional validation for annual change effects in independent longitudinal data (magenta), and the “1-stage Change” approach in longitudinal data (green). For each trait, the left panel shows power over varying genetic-by-age interaction or annual change effect size (varied from zero to 25% of a median marginal genetic effect on the trait; purple vertical dotted line denotes 10% of the median marginal effect) while keeping cross-sectional and longitudinal sample sizes constant at UKB configuration (**Table 1**). The right panel shows power of varying longitudinal-to-total sample size ratios (f = longitudinal sample size divided by the total sample size in UKB; assuming constant total sample size being the sum of cross-sectional and longitudinal sample size; the red vertical dotted line denotes the fraction of sample sizes given in UKB for the respective trait) while keeping the genetic effect constant at the 10% median marginal effect size from the left panel computation. Power was computed based on the analytical power formulae given in **Additional file 2:** **Note S2**, and assuming an allele frequency of 30%. Power computations for the other three traits are shown in **Figure 3**.


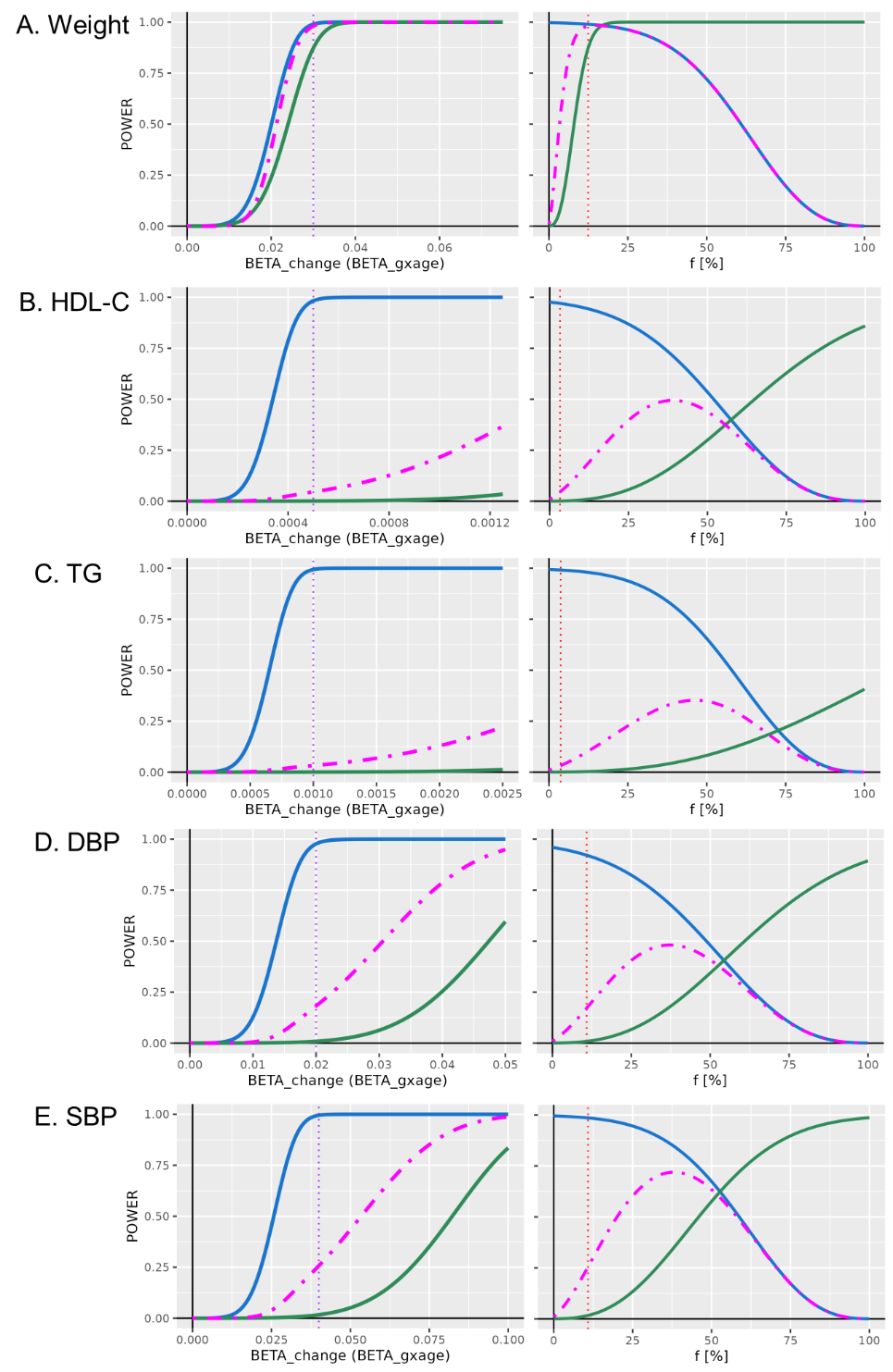


## **Fig S2. Miami plots comparing genetic-by-age interaction with marginal effect associations.**

Shown are the genome-wide Miami plots with genetic-by-age interaction P values on the upper side and marginal association P values on the lower side (based on cross-sectional data from UK Biobank excluding individuals with longitudinal data available; cross-sectional N >340,000). Significant genetic-by-age interaction loci (genome-wide significant: P_GxAge_<5x10^-8^; or 2-step significant: P_GxAge_<0.05/M_eff_, Bonferroni-corrected for the number of independent tests among all genome-wide significant marginally associated variants P<5x10^-8^) are coloured in blue and magenta with the latter being those that were validated for their genetic effects on annual trait change in longitudinal data (1-sided P_Change_<0.05/M_GxAge_, in independent longitudinal data from UKB, N up to 52,000). The Miami plots are shown for A: Weight, B: BMI, C: HDL-cholesterol, D: LDL-cholesterol, E: Triglycerides, F: Diastolic blood pressure, G: Systolic blood pressure, and H: Pulse pressure.


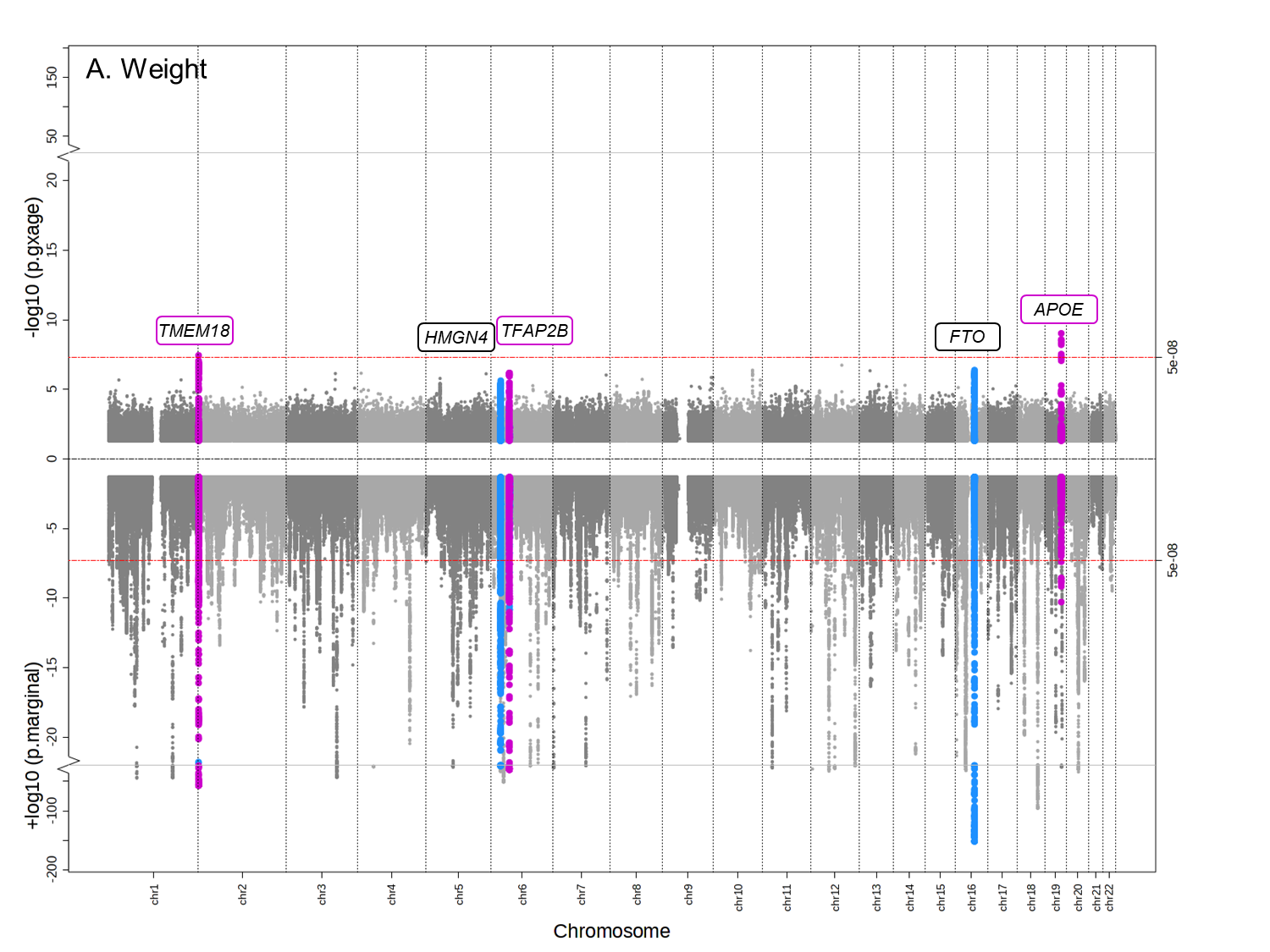


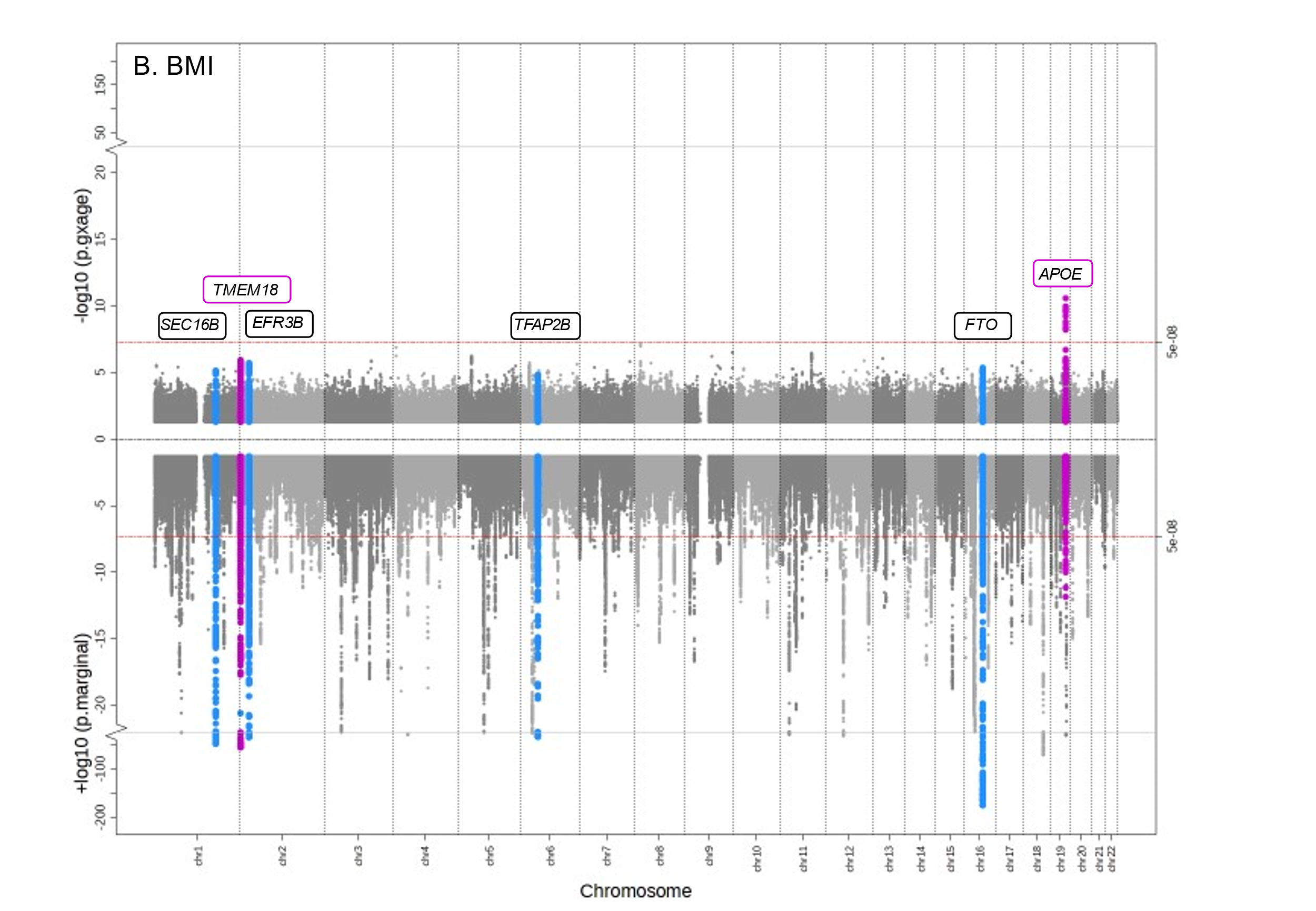

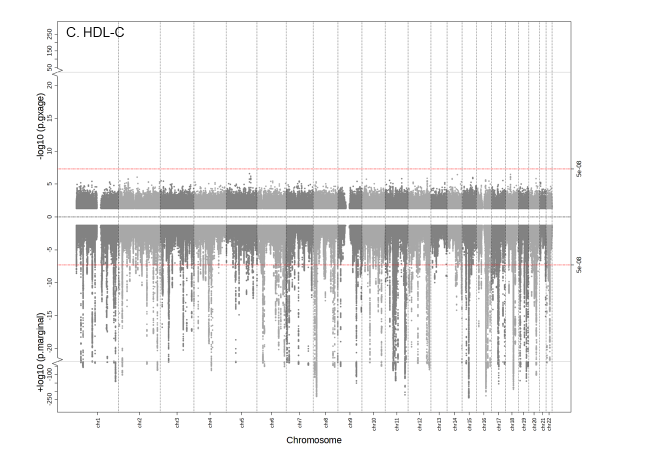

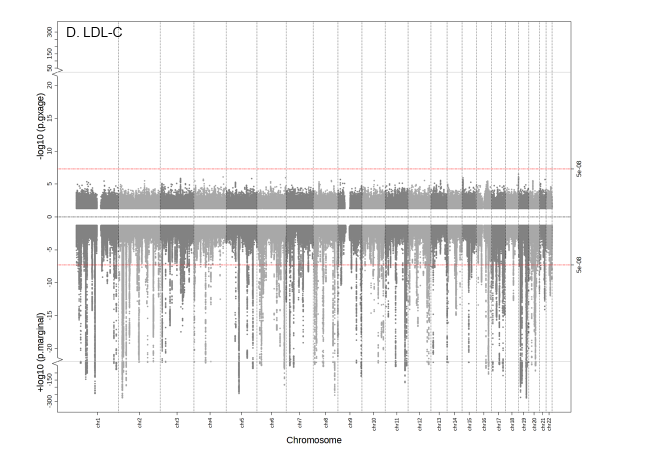

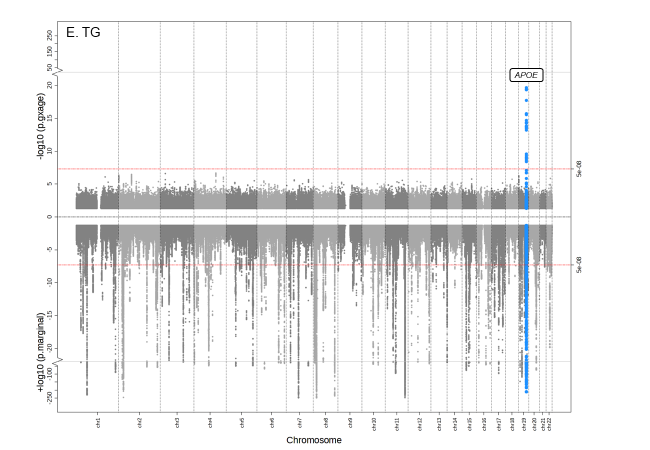

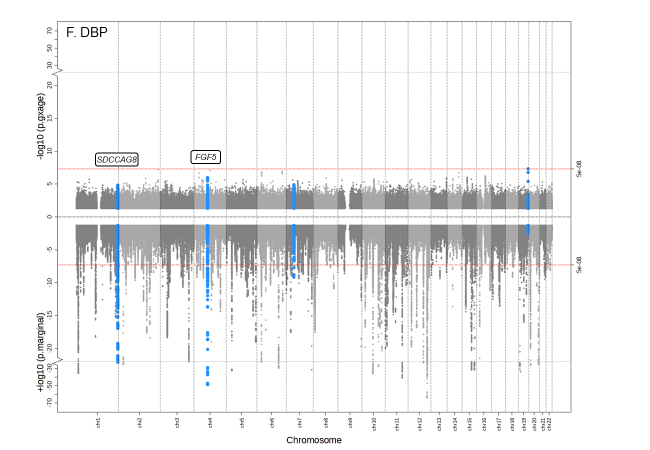

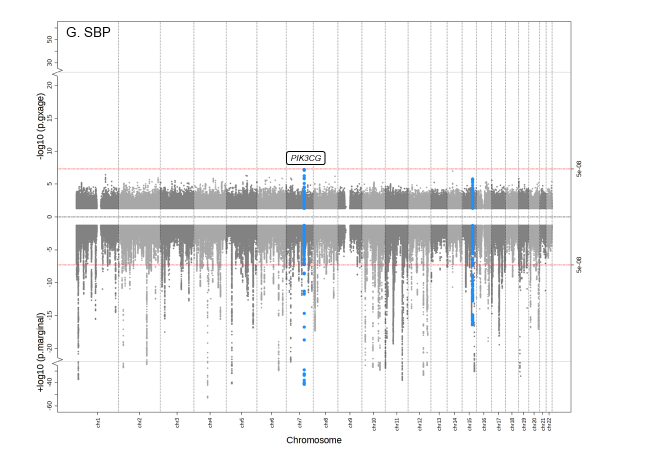

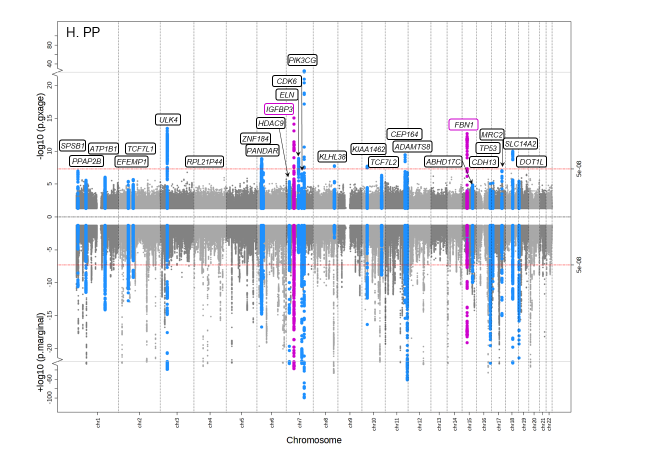


## **Fig S3. Heatmap of genetic-by-interaction effect sizes.**

Shown is a clustered heatmap for independent variants (r^2^<0.01) comparing their standardized genetic-by-age interaction effects on the eleven traits. Effect directions across 8 traits were aligned to positive genetic-by-age interaction effects on the trait that showed the significant genetic-by-age interaction (**Additional File 1:** **Table S4**). The 44 genetic-by-age interactions identified across traits included a number of variants/loci overlapping between traits. They were reduced to independent variants in the heatmap based on r^2^<0.01. For overlapping loci, the effect sizes were aligned to the variant with the smallest genetic-by-age interaction P-value. Naming of the rows consists of the chromosomal position, nearest gene, effect and other allele and trait for which the variant showed most significant genetic-by-age interaction.


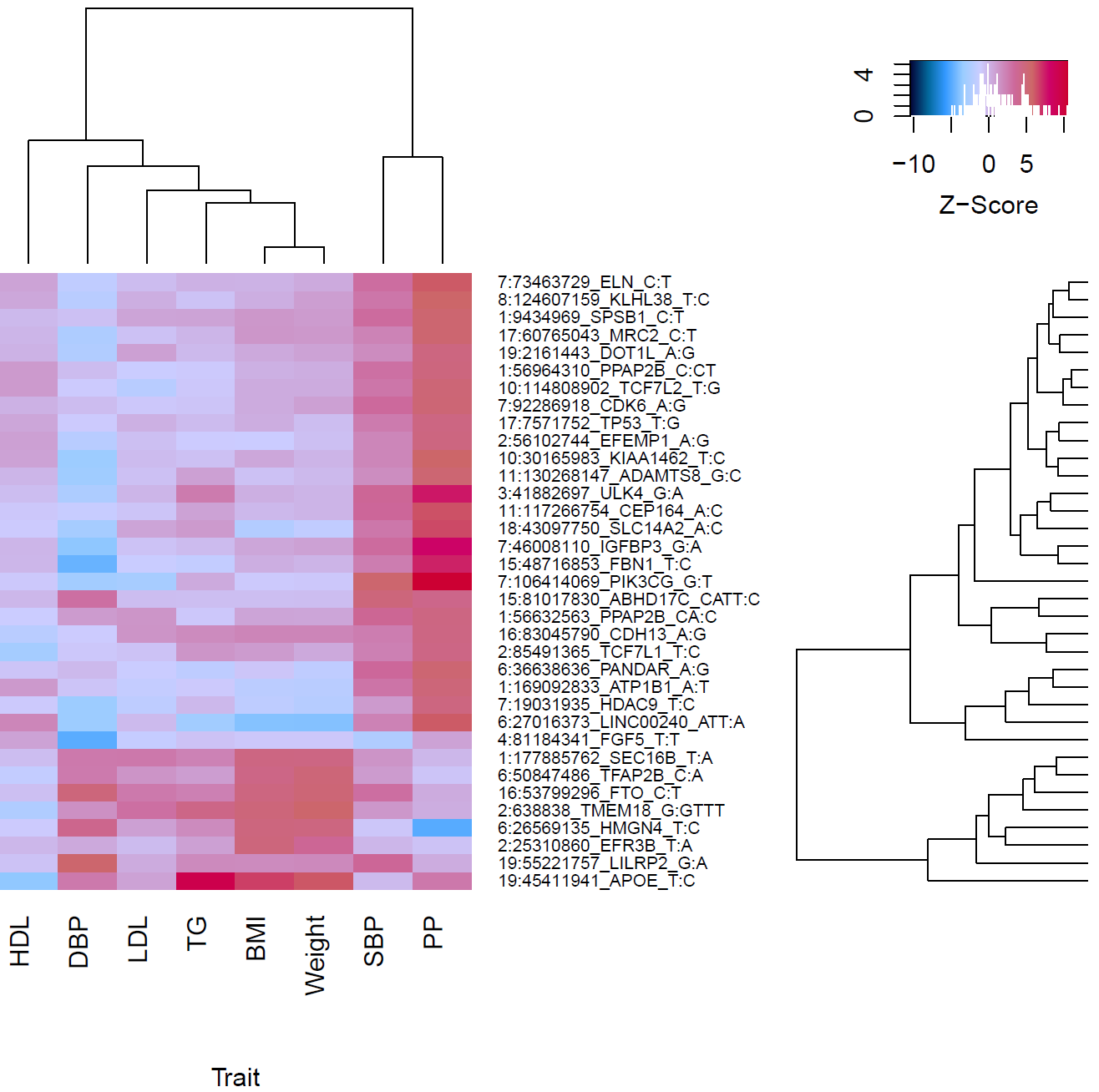


## **Fig S4. Comparison of gene-age interaction and marginal effect sizes.**

Shown are scatter plots comparing genetic-by-age interaction effect sizes (upscaled to 10 years, y axis) versus marginal genetic effect sizes (x axis) for the identified genetic-by-interaction variants (genome-wide significant: P_GxAge_<5x10^-8^; or 2-step significant: P_GxAge_<0.05/M_eff_, Bonferroni-corrected for the number of independent tests among all genome-wide significant marginally associated variants P<5x10^-8^). Magenta are those that were validated for their genetic effects on annual trait change in longitudinal data (1-sided P_Change_<0.05/M_GxAge_, in independent longitudinal data from UKB, N up to 52,000). The scatter plots are shown for A: Weight, B: BMI, C: TG-ln, D: Diastolic blood pressure, E: Systolic blood pressure, and F: Pulse pressure. All effect sizes were aligned to marginally trait-increasing alleles. **
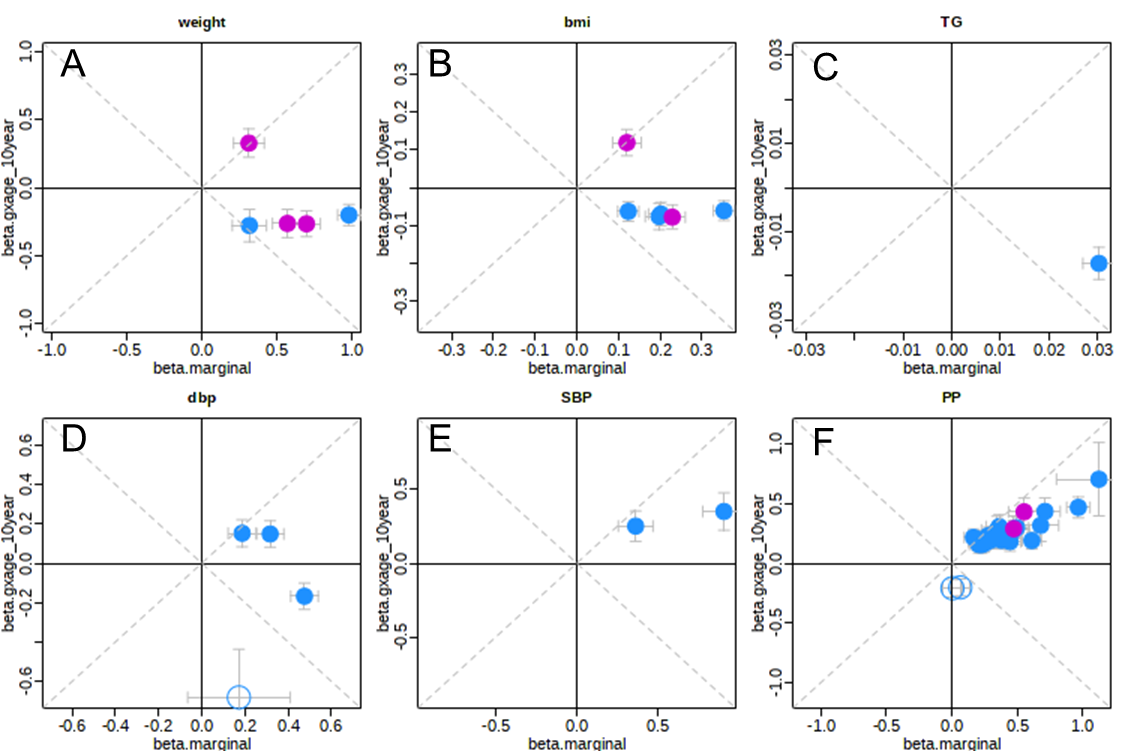
**

## **Fig S5. FUMA differentially up- and down-regulated expressed genes for pulse pressure loci with genetic-by-age interaction.**

Based on the 26 pulse pressure loci with significant genetic-by-age interaction (P_GxAge_<5x10^-8^ or significant in the 2-step approach), the figures show results from tissue-specific differentially expressed gene set enrichment analysis by FUMA (GENE2FUNC analysis; based 54 GTEx v8 tissue types; significant enrichments highlighted in red, FDR<5%). The results are shown for one-sided tests of enrichment of upregulated or downregulated differentially expressed genes. Results from the two-sided test are shown in **Figure 8.**


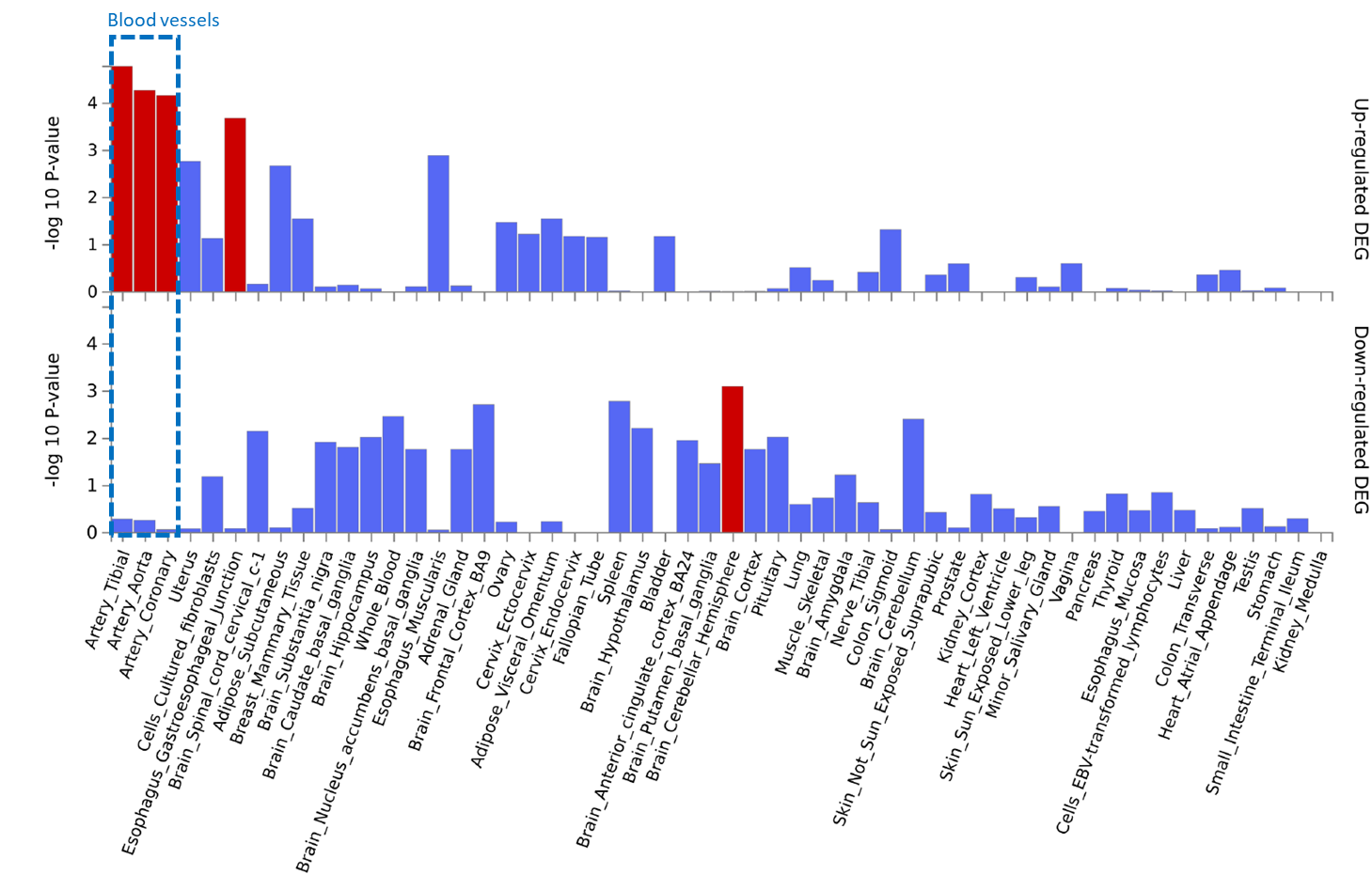


## **Fig S6. DEPICT tissue and cell-type specific enrichment at pulse pressure loci.**

Based on the 26 pulse pressure loci with significant genetic-by-age interaction (P_GxAge_<5x10^-8^ or significant in the 2-step approach, **Additional file 1:** **Table S3**), shown in figure A: are tissue and cell-type specific enrichment of gene expression analysis results by DEPICT (significant enrichments highlighted in blue, FDR<5%). For comparison, shown in B: are the DEPICT enrichment results for 26 pulse pressure loci without genetic-by-age interaction (i.e., P_Marginal_<5x10^-8^ and P_GxAge_>0.48, **Additional file 1:** **Table S5**).

**
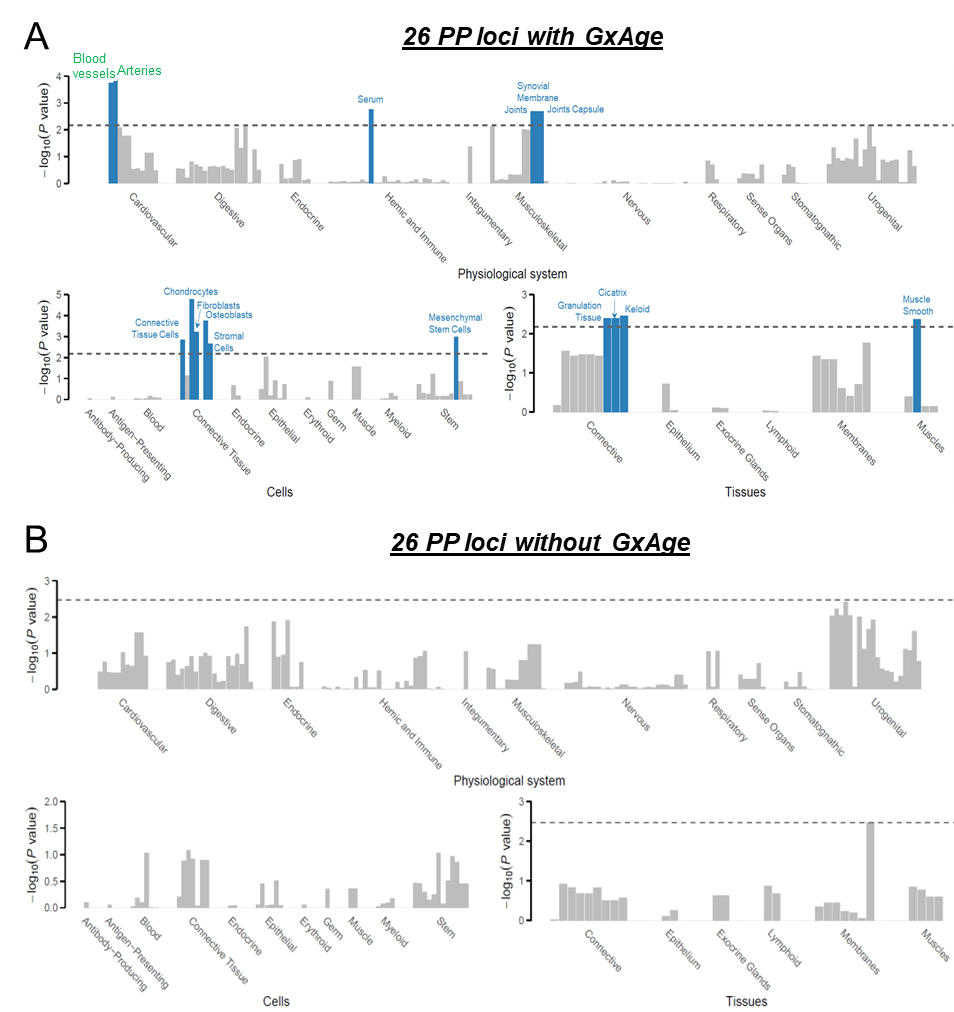
**

## **References**

1. Gorski M, Rasheed H, Teumer A, Thomas LF, Graham SE, Sveinbjornsson G, et al. Genetic loci and prioritization of genes for kidney function decline derived from a meta-analysis of 62 longitudinal genome-wide association studies. Kidney Int. 2022;102:624–39.

2. Behrens G, Winkler TW, Gorski M, Leitzmann MF, Heid IM. To stratify or not to stratify: Power considerations for population-based genome-wide association studies of quantitative traits. Genet Epidemiol. 2011;35:867–79.

3. Laville V, Bentley AR, Privé F, Zhu X, Gauderman J, Winkler TW, et al. VarExp: Estimating variance explained by genome-wide GxE summary statistics. Bioinformatics. 2018;34:3412–4.
