# Supplementary material for: Genetic-by-age interaction analyses on complex traits in UK Biobank and their potential to identify effects on longitudinal trait change
Source: Genome Biol. 2024 Nov 28;25:300. doi: 10.1186/s13059-024-03439-9 (PMC11606088; doi:10.1186/s13059-024-03439-9)
Supplement: Supplementary file 3 — Additional file 3. Review history. [file 13059_2024_3439_MOESM3_ESM.docx]

Review History

**First round of review**

**Reviewer 1**

**Were you able to assess all statistics in the manuscript, including the appropriateness of statistical tests used?**

Yes, and I have assessed the statistics in my report.

**Comments to author:**

The manuscript analyzes genetic-by-age interactions across multiple complex traits using UK Biobank data, along with a methodological exploration for identifying such interactions through cross-sectional and longitudinal data. This work contributes significantly to the fields of genomics and epidemiology, particularly in understanding the dynamic nature of genetic effects as they relate to age. The focus on longitudinal trait change is especially pertinent, addressing a critical gap in knowledge and offering insights for personalized medicine and targeted interventions. However, several areas of the manuscript could benefit from further refinement and clarification.
Comments
1. "We propose a new approach to exploit the relatively large cross-sectional data to prefilter genetic variants for genetic-by-age interaction", I do not think there is methodological innovation in the manuscript. The models used by the authors in the manuscript are conventional linear regression models.
2. "the assumption that there is no cohort effect", the author should propose a test to systematically validate the assumption in real dataset sets. This is important. I noticed that the authors did a sensitivity analysis for APOE. But this is limited.
3. I also would appreciate a more detailed discussion on the assumptions made, such as the lack of cohort effect and linear age effect on the traits. Additionally, the sensitivity analysis, including "birth year" as a covariate, is commendable, but it would be beneficial to discuss any potential limitations of this approach.
4. It would be interesting to extend the framework to incorporate both cross-section and longitudinal data.
5. As the linear regression is mathematically mature, I would suggest the authors provide a math formula to power besides the simulation experiments.
6. Moreover, the sample size ratios of the three different study designs may also be interesting.
7. Theoretically, a variant with genetic-by-age interaction will marginally affect the trait, although not all trait-affecting variants have genetic-by-age interaction. It will be important to systematically explore the proportions of the variants with genetic-by-age interaction and the effect direction.
8. Based on the proportions, some desmostration can be made to guide associated analysis and heritability estimation for GWAS.
9. The power comparison must systematically consider the sample size ratio. Figure 3 is not enough. By the way, the figure is also hard to follow. The legend should be integrated into the plots.
10. "The identified genetic-by-age interaction effects mostly overlapped with marginal effect loci.." This is expected. It would be necessary to systematically compare the involved biological pathways associated with variants with and without the genetic-by-age interaction effect. The authors provide a more detailed discussion of the biological relevance of the identified loci and their potential functional implications. Is the difference on variant level or gene levels?
11. The discussion could be enhanced by comparing the findings with previous studies and discussing the potential reasons for the observed differences in genetic-by-age interactions across traits.
12. The authors acknowledge the limitations of their study, such as the focus on the European population and the use of chronological age. It would be beneficial to discuss how these limitations might affect the generalizability of the findings and any potential strategies to overcome them in future research.

**Reviewer 2**

**Were you able to assess all statistics in the manuscript, including the appropriateness of statistical tests used?**

Yes, and I have assessed the statistics in my report.

**Comments to author:**

The authors describe and conduct a 2-stage approach for detecting genetic-by-age interactions, which includes a pre screening step and an independent longitudinal analysis. While the presented analyses are performed across a variety of phenotypes with abundant data and visualizations, I have a few comments:

Major comments:

-Certain metrics (e.g. BMI) may increase over age. Is it possible to correct for e.g. BMI or other covariates that might partly explain the strong relationship between pulse pressure and age? How have you taken this into consideration (this was not clear from initial reading)?

-"The fact that genetic effect sizes on kidney function and pulse pressure are enlarged at older ages underscores that the identified loci predispose accelerated aging processes" Can you relate this comment to the literature on epigenetic aging/DNAm age clocks, which are a complementary method to understand the relationship between aging and different diseases/traits? Can you also relate/discuss observations re pulse pressure and other phenotypes to specific aging processes (e.g. myeloid-biased output haematopoietic stem cells -my-HSCs, https://www.nature.com/articles/s41586-024-07238-x).

-it is hard to link many variants to genes, and therefore gene-based analyses of GWAS data can cause a loss in variant-to-tissue signal. To address this issue, can you show a direct variant-to-tissue chromatin activity method (e.g. FORGE2 https://forge2.altiusinstitute.org/) and its tissue-specific associations across pulse pressure and other phenotypes (this comment is related to "Figure 8. Tissue-specific enrichment of gene expression at genetic-by-age interaction loci for pulse pressure").

-GWAS tissue enrichment analysis should be reported not just for pulse pressure, but across all traits with associations (not necessary for the main figure set, but some follow up analysis across traits should at least be shown in the supplement).

-analysis code should be put on a github repository or in zenodo (ideally with a short tutorial)

Minor comments:

line 160: "describe the results in the following." should be "describe the results in the following section."

line 330: "Alzheimer" -> "Alzheimer's".

**Authors Response**

**Point-by-point responses to the reviewers’ comments:**

# **Reviewer #1:**

The manuscript analyzes genetic-by-age interactions across multiple complex traits using UK Biobank data, along with a methodological exploration for identifying such interactions through cross-sectional and longitudinal data. This work contributes significantly to the fields of genomics and epidemiology, particularly in understanding the dynamic nature of genetic effects as they relate to age. The focus on longitudinal trait change is especially pertinent, addressing a critical gap in knowledge and offering insights for personalized medicine and targeted interventions. However, several areas of the manuscript could benefit from further refinement and clarification.

***Response:*** *We thank the reviewer for the very clear and constructive comments. We have addressed the comments below point-by-point and revised the manuscript accordingly (we marked all added and revised chapters and items in yellow in the manuscript).*

*In addition to the requested changes, we have decided to remove the kidney traits from this paper. The main reason for this was that kidney traits were the only one to use data outside of UK Biobank, which made the analysis workflow a little asymmetric and overly complex. There were also no novel findings for kidney. Given the additional analyses conducted for the revision, we decided to remove kidney traits in order to focus the paper on the interesting aspects while keeping the analyses symmetric.*

Comments
1. "We propose a new approach to exploit the relatively large cross-sectional data to prefilter genetic variants for genetic-by-age interaction", I do not think there is methodological innovation in the manuscript. The models used by the authors in the manuscript are conventional linear regression models.

***Response:*** *We agree with the reviewer that the regression models are conventional linear regression models. Yet, we think that this is the first report that describes the ability of cross-sectional gene-age interaction testing to reduce multiple testing burden (thus increase power) for the longitudinal genetic association testing. Such 2-step approaches are known for gene-environment interaction testing in (non-longitudinal) data where a first step is utilized to preselect variants and then a second step is utilized to test the selected variants for GxE at a reduced multiple testing burden (e.g., PMID: 23843674, 22714933, 36571162). We consider the combination of the two stages/steps as “integrated approach” but agree that this was not described clearly. We have thus reworded the sentence as follows (p. 3):*

*Here, we propose a 2-stage approach to gain power in testing for genetic effects on longitudinal trait change. The approach comprises a first stage to select variants with significant genetic-by-age interaction in cross-sectional data and a second stage to test the selected variants for association with trait-change in longitudinal data. The 2-stage approach reduces the multiple testing burden in the longitudinal data by exploiting relatively large cross-sectional sample sizes, and thus power, in the first stage.*

2. "the assumption that there is no cohort effect", the author should propose a test to systematically validate the assumption in real dataset sets. This is important. I noticed that the authors did a sensitivity analysis for APOE. But this is limited.

***Response:*** *We agree with the reviewer that lack of cohort effect is important in longitudinal data analysis. In the previous version of the paper, we have addressed this by evaluating the identified GxAge effects after adjustment for birthyear (to evaluate potential cohort confounding by birthyear effects) and tested the identified GxAge variants for association with age as the outcome (which we expected to pinpoint cohort effects in UKB). Based on the reviewer comment, we have extended our analyses to testing the identified GxAge variants for association with birthyear as the outcome. The reason for this is because there is an important distinction between age and birthyear: SNP associations on age pinpoint effects on longevity or age-dependent selection into the study, while SNP associations on birthyear pinpoint cohort effects depending on birthyear. Importantly, in a cohort study like UKB, where all* *individuals attend the study center in a relatively short time span of few years, age and birthyear will be highly correlated (r=-0.99 in UKB), so that the distinction and interpretation of pinpointed effects will be less obvious. To address this, we have now also conducted SNP association tests on birthyear, and updated Supplementary* ***Table S6*** *(which now combines all three sensitivity analysis results for all identified GxAge variants in a single table):*

*We have extended the description of the sensitivity analyses in the main text as follows (p. 9):*

*To investigate whether cohort, selection or survival effects confounded the observed genetic-by-age interaction effects, we conducted three sensitivity analyses (****Methods****). To evaluate cohort effects that may stem from birthyear effects on genetics and outcomes, we first included birthyear as covariate in the genetic-by-age interaction regression model and observed stable genetic-by-age interaction effects for all variants (****Table S6****). We then tested the variants for association with birthyear as the outcome in a linear regression model and observed significant effects on birthyear for the APOE variant rs429358 (P_G🡪Birthyear_=2.6x10^-5^,* ***Table S6****). To evaluate selection and survival effects, we tested for association of the variants with age and again found one significant age-association for the APOE variant rs429358 (P_G🡪Age_=4.6x10^-5^,* ***Table S6****). Due to the high correlation between birthyear and age in UKB (r=-0.99; due to recruiting within a relatively short time span of few years) and the known detrimental effects of APOE on health, we concluded that the observed genetic-by-age interactions of APOE on BMI, weight and triglycerides were likely confounded by age-dependent selection of individuals into UKB rather than cohort effects that would stem from birthyear effects. Given the theoretical similarity of genetic-by-age and genetics-of-change effect sizes shown above, our sensitivity analyses underscore that our identified variants - except APOE - are truly affecting longitudinal change of the respective traits.*

*We describe the proposed strategy and limitations in the discussion (p. 15):*

*Importantly, the identified genetic-by-age interaction and annual change effects can be biased due to confounding by cohort effects, i.e., birthyear, or by age-dependent selection/survival effects. Cohort effects can be addressed by adjusting the genetic-by-age interaction effects for birthyear and by testing variants for association with birthyear. Selection/survival effects can be addressed by testing variants for association with age. Importantly, for cohort studies like UKB that typically recruit individuals in a relatively short time span of few years, birthyear and age will be highly correlated, which is why similar age and birthyear variant associations are to be expected. Thus, their results should be carefully interpreted in the light of known mechanisms for the specific variant. Among our identified variants, we observed significant effects on birthyear and age for the APOE variant rs429358, which is also known to be associated with Alzheimer’s and early mortality [11–13]. For the risk-increasing allele, we observed a significant effect on decreased age, which is in line with more frequent allele frequency of the risk allele in the younger and thus age-dependent selection/survival bias in the older-aged participants of UKB. This suggests confounding by age-dependent selection/survival rather than birthyear cohort effects for APOE.*

*We have also added a new methods chapter on the sensitivity analyses (p. 21):*

## Sensitivity analyses for cohort, selection and survival effects

*To avoid confounding of identified genetic-by-age interaction and annual change effects by cohort, selection or survival effects, we conducted the three following sensitivity analyses in unrelated European individuals of UKB. (i) For cohort effects, we first fitted the genetic-by-age interaction model while adjusting for birthyear:*

$$Y=\beta_{0}+\beta_{G}G+\beta_{Age}AGE+\beta_{GxAge}G\cdot AGE+\beta_{BY}BY+\beta_{C}C+\varepsilon$$

*Here, Y is the trait value, G the allelic dosage, AGE the age centered at the mean age of all individuals, BY is the birthyear, and C is a matrix of further covariates. We obtained genetic-by-age interaction effects and compared them to the effects from the BY-unadjusted interaction model assuming that differences between the models would pinpoint potential confounding by cohort. (ii) For cohort effects, we also fitted a linear regression model of the genetic variants on birthyear:*

$$BY=\beta_{0\to BY}+\beta_{G\to BY}G+\beta_{C\to BY}C+\varepsilon_{BY}$$

*We obtained genetic effects of the variants on birthyear assuming that significant birthyear associations would pinpoint potential cohort effects. (iii) similarly, for age-dependent selection and survival, we fitted a linear regression model of the genetic variants on age:*

$$AGE=\beta_{0\to AGE}+\beta_{G\to AGE}G+\beta_{C\to AGE}C+\varepsilon_{AGE}$$

*We obtained genetic effects of the variants on age assuming that significant age associations would pinpoint confounding by age-dependent selection or survival.*

3. I also would appreciate a more detailed discussion on the assumptions made, such as the lack of cohort effect and linear age effect on the traits. Additionally, the sensitivity analysis, including "birth year" as a covariate, is commendable, but it would be beneficial to discuss any potential limitations of this approach.

***Response:*** *Indeed,* *the assumptions are important in longitudinal data analysis. The linear age effect is a prerequisite for the analytical equivalence of genetic-by-age interaction and the longitudinal trait change effect. We want to note that linear genetic effects (or interactions) in GWAS are a common assumption/limitation in GWAS. The linear assumption will catch some non-linear effects but not all.*

*We have extended the discussion on cohort effects as shown for the previous comment and furthermore added the following sentence on linearity to the discussion (p. 16):*

*First, the genetic-by-age interaction screening assumes a lack of age effect on the outcome difference between two time points, i.e., linear age effect on the outcome, and thus will miss or have reduced power to identify non-linear age effects.*

*We address the following three comments on power in one response:*

5. As the linear regression is mathematically mature, I would suggest the authors provide a math formula to power besides the simulation experiments.

6. Moreover, the sample size ratios of the three different study designs may also be interesting.

9. The power comparison must systematically consider the sample size ratio. Figure 3 is not enough. By the way, the figure is also hard to follow. The legend should be integrated into the plots.

***Response:*** *We completely agree with the reviewer that additional content on power will be helpful and interesting for the readers. Considering sample size ratios helps the interpretation of the power.*

*As requested by the reviewer, we have*

(i) *derived analytical power formulae for the three approaches (‘1-stage GxAge approach’, ‘1-stage Change approach’ and ‘2-stage GxAge->Change approach’) and have added the derivation and the formulae in the new Note S2.*
(ii) *extended existing power computations and their description in the main text to traits other than BMI. We have added a new Table 2 (see below) that shows power and minimum detectable effect size for all traits and have revised and extended the description in the results chapter (see below).*
(iii) ***updated*** ***Figure 3*** *as requested. We have removed the differentiation between genome-wide significance and 2-step tests and now only show the three aggregated power curves for the three approaches. We have added a legend describing the curves. The previous figure was limited to assumptions based on BMI. We have added sub-Figures for LDL-C and pulse pressure.*
(iv) *systematically considered the sample size ratios (f=Nlong/Nnolong) in the power computations and have added respective sub-figures to* ***Figure 3****. We demonstrate that for BMI and weight the “1-stage Change” approach overcomes the other approaches (involving GxAge testing) at f~25% (i.e., ~92,000 individuals with longitudinal data; which is almost twice as much as would be available for BMI or weight in UKB). For all other traits, the “1-stage Change” approach cannot overcome the other approaches, even when longitudinal sample sizes were identical to the respective cross-sectional sample size (f=1). The reason for this is that annual change effects for these traits explain a relatively lower fraction of the change variance (****new Table 2****), which is why their identification requires much higher sample sizes.*

*Like Figure 3, we have extended* ***Figure S1*** *that shows power results for the other traits.*

*We have revised the description of the power computations in the main text results as follows (p 6):*

*We first compared the power of the three approaches for varying effect sizes and for sample size configurations that reflect UKB (cross sectional N>350,000 and longitudinal N<50,000). We observed a substantial improvement in power to identify genetic effects on trait change by the approaches involving genetic-by-age interaction testing (left panels in* ***Figure 3****,* ***Figure S1****). For example, assuming a realistic genetic effect on annual trait change that is equal to 10% of a median marginal effect for the trait (i.e., the mean genetic effect size is modulated each year by 10%), we observe >91% power for the “1-stage GxAge” approach across all traits (****Table 2****). Power of the change approaches for this 10% effect was reasonable for weight and BMI but extensively attenuated for the other traits (****Table 2****). The reason for this was that the 10% effect explained more variance for annual change of BMI (~0.05%, for an allele frequency of 30%) as compared to annual change of other traits (e.g., only 0.004% and 0.009% variance explained for change of LDL-C and pulse pressure, respectively,* ***Table 2****). This pattern is confirmed by calculations of minimum annual change effect size detectable at 80% power for the three approaches (****Table 2****). While all approaches capture realistic annual weight- and BMI change effects, only the “1-stage GxAge” approach captures realistic annual change effects for the other traits (****Table 2****). The minimum effect sizes detectable by the change approaches were unrealistically high (e.g., 0.47 (mg/dl)/year/allele for LDL-C, which refers to a yearly modulation that is equivalent to 59% of a median marginal effect).*

*We next compared the power of the three approaches for varying ratios of longitudinal to cross sectional sample sizes while keeping the annual change effects constant at the minimum effect detectable at 80% by the “1-stage GxAge” (right panels in* ***Figure 3****,* ***Figure S1****). We varied the longitudinal sample size from 0 (proportion of longitudinal to cross-sectional N, f=0) up to the assumed cross-sectional sample size (f=1). For BMI, we found that the “1-stage Change” approach overcame the other two approaches at f~25%, which reflects a longitudinal sample size of ~92,000 individuals compared to 370,000 individuals cross-sectionally. For all other traits (except weight), the “1-stage Change” approach cannot overcome the other approaches, even when longitudinal sample sizes were identical to the respective cross-sectional sample size (f=1). Again, the reason for this is that annual change effects for these traits explain a relatively lower fraction of the change variance, which is why their identification requires much higher sample sizes.*

*We have revised* ***Figure 3*** *as follows:*

## Figure 3. Power to identify genetic-by-age interaction and longitudinal change effects in UKB.

*Shown are power curves for genetic-by-age interaction and annual change effects on A: BMI, B: LDL-C and C: PP. Power is shown for the three approaches: the 1-stage GWAS on GxAge approach in cross-sectional data (blue), the 2-stage GWAS on GxAge🡪Change approach that includes additional validation for annual change effects in independent longitudinal data (magenta), and the 1-stage GWAS on change approach in longitudinal data (green). For each trait, the left panel shows power over varying effect size* *(varied from zero to 25% of a median marginal genetic effect on the trait; blue vertical dotted line denotes the genetic-by-age interaction effect at which power of the 1-stage GWAS on GxAge approach is exactly 80%; orange vertical dotted line denotes 10% of the median marginal effect) while keeping cross-sectional and longitudinal sample sizes constant. The right panel shows power of varying longitudinal-to-cross-sectional sample size ratios (f = longitudinal sample size divided by the given cross-sectional sample size in UKB; the red vertical dotted line denotes the fraction of sample sizes given in UKB for the respective trait) while keeping the genetic effect constant at the 80% power effect size from the left panel computation. Power was calculated for an allele frequency of 30%, based on analytical formulas given in* ***Note S2*** *and assumptions given in* ***Methods****. Power computations for the remaining traits are shown in* ***Figure S1****.*

*We have added the following* ***new Table 2****:*

7. Theoretically, a variant with genetic-by-age interaction will marginally affect the trait, although not all trait-affecting variants have genetic-by-age interaction. It will be important to systematically explore the proportions of the variants with genetic-by-age interaction and the effect direction.

***Response:*** *Absolutely, this is what we have tried previously by enrichment analysis, which however did not account for the directionality between marginal and interaction effects. Based on the reviewer’s suggestion, we have added the proportions to the* ***updated*** ***Table 3*** *(see below) and now consider the direction. We found a significant enrichment of intensified effects for PP and SBP, and a significant enrichment of attenuated effects for BMI, weight and LDL-C. We have updated the* *main text as follows (p. 9):*

*Vice versa, starting from trait-specific genome-wide significant marginal effect lead variants (marginal P<5x10^-8^, d>500kb,* ***Table S5****), we observed a significant intensification of effects with increasing age for systolic blood pressure and pulse pressure, and a significant attenuation of effects for weight, BMI and LDL-cholesterol (enrichment P_binom_<0.05/8, Bonferroni corrected for 8 traits* ***Table 3****). On average, the yearly modulation of genetic effect size ranged between 3.2%-4.8% of the median marginal effect size across traits. The strongest enrichment was observed for pulse pressure where intensified effects were observed for 101 (56.4%) of the 179 marginally associated region lead variants. This trait-specific enrichment provides the degree of the trait’s genetics that is age-dependent and its direction.*

*We have* ***updated*** ***Table 3*** *as follows:*

*We have also added this important aspect to the abstract (p 2):*

*Our cross-trait view revealed trait-specificity regarding the proportion of loci with age-modulated effects, which was particularly high for pulse pressure.*

*and to the discussion (p 13):*

*Considering a broader spectrum of marginally associated trait variants confirmed these observed proportions and directions, with >50% of pulse pressure variant effects being intensified, and ~15% of obesity variant effects being attenuated with age.*

8. Based on the proportions, some demonstration can be made to guide associated analysis and heritability estimation for GWAS.

***Response:*** *We fully agree with the reviewer that our findings may inform associated GWAS analyses. However, these will be manifold with different implications on various aspects of GWAS follow up. We think that a comprehensive illustration of how to account for age dependency in these analyses will be too extensive for this paper. Yet, to make readers aware of this methodological potential, we have added the following text to the discussion (p 17):*

*Our results emphasize the importance to account for age-dependency in several aspects of GWAS, such as fine mapping or heritability estimation, and may open the route to future methods developments.*

10. "The identified genetic-by-age interaction effects mostly overlapped with marginal effect loci." This is expected. It would be necessary to systematically compare the involved biological pathways associated with variants with and without the genetic-by-age interaction effect. The authors provide a more detailed discussion of the biological relevance of the identified loci and their potential functional implications. Is the difference on variant level or gene levels?

***Response:*** *We thank the reviewer for this important suggestion. Based on this comment and complimentary to the existing tissue enrichment at GxAge loci, we have added tissue-specific enrichment analyses for main effect loci that do not change their effects with age (no-GxAge). For pulse pressure, we found a clear distinction of tissue enrichments between GxAge and no-GxAge loci. While* *GxAge loci picked up genes with expression effects in relevant blood vessel tissues, this was not observed for the no-GxAge loci. The finding was consistently observed in enrichment analyses by FUMA, DEPICT and FORGE2. The latter is a direct variant-to-tissue method based on chromatin activity, which was added based on one comment by Reviewer 2. We thus concluded that the difference derives more from genes being involved in aging processes rather than the variants themselves. We present these comparative analyses in the updated main text, the* ***updated Figure 8*** *(panel B and C show the FUMA results for GxAge and no-GxAge loci, respectively), the* ***new Figure S6*** *(panel A and B show the DEPICT results for GxAge and no-GxAge loci, respectively) and* ***new Table S11*** *(FORGE2 results for GxAge and no-GxAge loci).*

*The updated main results text reads as follows (p 12):*

*We conducted tissue-specific expression analyses with FUMA and observed significantly differentially expressed genes in tibial arteries and in aorta arteries (FDR<5%,* ***Figure 8A+B****,* ***Table S9****). In terms of direction, the enrichment in arteries was particularly observed for upregulated gene expression (****Table S9, Figure S5****). A confirmatory pattern was observed by enrichment analysis with DEPICT, which yielded significant enrichment of gene expression effects in 16 tissues and cell types including blood vessels, arteries, veins, and endothelial cells (tissue enrichment FDR<5%,* ***Figure 8C, Figure S6, Table S10****). Strikingly, the enrichment in relevant tissues could not be found in complimentary enrichment analyses that were based on 26 pulse pressure loci without genetic-by-age interaction (i.e., associated but no genetic-by-age interaction; P_Marginal_<5x10^-8^ and P_GxAge_>0.48;* ***Figure 8B+C****).* *This distinction was confirmed by direct variant-to-tissue mapping based om chromatin activity (FORGE2 [14]), which yielded a significant enrichment of blood vessel cells for the 26 genetic-by-age interaction pulse pressure loci (4 out of 14 blood vessel cells were mapped with nominal significance, binomial enrichment P= 0.004) but no enrichment for the 26 loci without genetic-by-age interaction (****Table S11****). Together, these results underscore the biological relevance of age as intensifying modulator in pulse pressure genetics and biology.*

*The updated* ***Figure 8*** *looks as follows:*

*The new* ***Figure S6*** *with DEPICT results looks as follows:*

*The new* ***Table S11*** *with FORGE2 results looks as follows:*

4. It would be interesting to extend the framework to incorporate both cross-section and longitudinal data.

***Response:*** *We fully agree with the reviewer that combining cross-sectional and longitudinal data into a single linear mixed model would be ideal. However, this is currently difficult to apply for GWAS. There is no LLM implementation for GWAS of biobank-scale data available that allows to integrate longitudinal data with GxAge testing, and to incorporate individuals with single measurements. Furthermore, this model may strongly depend on the sample size ratios of individuals with single versus multiple measurements, as well as on the number of trajectories per person. In our situation, we have approx. 350,000 individuals with single measurements and only up to 50,000 with two measurements. It is not clear, how LMMs with person-specific intercepts and slopes will perform in this situation. To address this, we have added the following text to the discussion (p 16):*

*Furthermore, while LMMs would generally allow to simultaneously analyze individuals with single and with multiple measurements, its performance and methodological validity are unclear to date and may depend on the proportion of individuals with single versus multiple measurements.*

11. The discussion could be enhanced by comparing the findings with previous studies and discussing the potential reasons for the observed differences in genetic-by-age interactions across traits.

***Response:*** *Generally, findings of genetic-by-age interaction for the metabolic traits are sparse. To our knowledge, there is no previous work that included GxAge terms in GWAS regression models. Yet, for BMI and blood pressure traits including pulse pressure, we found two large age-stratified GWAS meta-analyses by GIANT (for BMI) and by CHAREG/GBPgen/ICBP for blood pressure. For BMI, four of our loci overlapped with the age-specific loci by GIANT. For pulse pressure, only one locus (near PIK3CG) was mentioned for its nominal significant age-dependent effect by the previous paper (not by conservative Bonferroni-corrected significance).*

*We address these papers in the discussion (p. 13):*

*This pattern was consistent with previous work from the GIANT consortium that identified 11 loci with smaller genetic effect sizes on BMI among older compared to younger adults using age-stratified GWAS meta-analyses on cross-sectional data [15]. Four of the 11 loci by GIANT were validated by our analyses of BMI-change using independent longitudinal data from UKB (near TMEM18, EFR3B, SEC16B and FTO).*

*We mention the previous blood pressure paper also in discussion (p 14):*

*Only the locus near PIK3CG was mentioned before for a potential age-dependent effect on pulse pressure, however without Bonferroni-corrected significance [28].*

12. The authors acknowledge the limitations of their study, such as the focus on the European population and the use of chronological age. It would be beneficial to discuss how these limitations might affect the generalizability of the findings and any potential strategies to overcome them in future research.

***Response****: Indeed, these are important points for the reader to put our results into context of future work. Our framework can easily be generalized and be applied to other ancestries and traits. We address this in discussion (p 17):*

*Another limitation of our study is the focus on the European population. Yet, our 2-stage approach is readily applicable to non-European populations and to other traits [34].*

*In terms of chronological vs biological age, we now provide a more detailed discussion on the interpretation of gage-dependent effects on the molecular level (p. 17):*

*Biological aging can differ from chronological aging at the molecular level. Age clocks based on telomere length and age-dependent DNA methylation CpG sites reflect this process [37]. Also, the aging immune system, the balance of antibody-producing cells and T cells is strongly age-dependent and has an impact on the ability to respond to several infections [38]. Thus, any observed genetic-by-age interaction could potentially be explained by genetic-by-CpG interactions or be linked to mechanisms that are related to the age-dependent immune system*.

# **Reviewer #2:**

The authors describe and conduct a 2-stage approach for detecting genetic-by-age interactions, which includes a pre screening step and an independent longitudinal analysis. While the presented analyses are performed across a variety of phenotypes with abundant data and visualizations, I have a few comments:

***Response:*** *We thank the reviewer for the thorough review, which helped us to improve the manuscript. We have addressed the comments below point-by-point and revised the manuscript accordingly (we marked all added and revised chapters and items in yellow in the manuscript).*

*In addition to the requested changes, we have decided to remove the kidney traits from this paper. The main reason for this was that kidney traits were the only one to use data outside of UK Biobank, which made the analysis workflow a little asymmetric and overly complex. There were also no novel findings for kidney. Given the additional analyses conducted for the revision, we decided to remove kidney traits in order to focus the paper on the interesting aspects while keeping the analyses symmetric.*

Major comments:

1. Certain metrics (e.g. BMI) may increase over age. Is it possible to correct for e.g. BMI or other covariates that might partly explain the strong relationship between pulse pressure and age? How have you taken this into consideration (this was not clear from initial reading)?

***Response:*** *We appreciate the reviewer’s feedback. We concur that any GxAge interaction identified could potentially reflect the interaction of the SNP with another age-dependent variable, such as BMI (GxAge might indeed be GxBMI). The reason GxAge was detected may be because GxBMI was not included in the model as a covariate. It’s important to recognize that confounding of GxE by GxC can occur when E and C are correlated. To address this issue, we have performed sensitivity analyses by incorporating GxBMI interaction terms as covariates in the GxAge interaction model for pulse pressure. The results are detailed in the main text (p. 12):*

*The observed genetic-by-age interaction effects for pulse pressure were not confounded by genetic-by-BMI interaction in sensitivity analyses (****Table S18****).*

*The sensitivity results were added to the* ***new Table S8****:*

2. "The fact that genetic effect sizes on kidney function and pulse pressure are enlarged at older ages underscores that the identified loci predispose accelerated aging processes" Can you relate this comment to the literature on epigenetic aging/DNAm age clocks, which are a complementary method to understand the relationship between aging and different diseases/traits? Can you also relate/discuss observations re pulse pressure and other phenotypes to specific aging processes (e.g. myeloid-biased output haematopoietic stem cells -my-HSCs, https://www.nature.com/articles/s41586-024-07238-x).

***Response:*** *We completely agree with the reviewer on this aspect. While the observed impact of age on the genetic effects on pulse pressure seems clear and robust, its biological mechanisms on the molecular level, i.e., how age impacts the genetic effects, are to be studied further. Indeed, given the known connection between methylation and aging, one explanation could be that age dependent CpG sites impact the genetic effects on pulse pressure, i.e., GxAge mirrors potential GxCpG effects. We have added this important aspect to the discussion as follows (p 17):*

*Biological aging can differ from chronological aging at the molecular level. Age clocks based on telomere length and age-dependent DNA methylation CpG sites reflect this process [36]. Also, the aging immune system, the balance of antibody-producing cells and T cells is strongly age-dependent and has an impact on the ability to respond to several infections [37]. Thus, any observed genetic-by-age interaction could potentially be explained by genetic-by-CpG interactions or be linked to mechanisms that are related to the age-dependent immune system.*

3. it is hard to link many variants to genes, and therefore gene-based analyses of GWAS data can cause a loss in variant-to-tissue signal. To address this issue, can you show a direct variant-to-tissue chromatin activity method (e.g. FORGE2 https://forge2.altiusinstitute.org/) and its tissue-specific associations across pulse pressure and other phenotypes (this comment is related to "Figure 8. Tissue-specific enrichment of gene expression at genetic-by-age interaction loci for pulse pressure").

***Response:*** *We thank the reviewer for the suggestion and completely agree that a direct variant-to-tissue method will complement our tissue enrichment analyses (which require an intermediate gene mapping step). We have applied the FORGE2 method to our 26 genetic-by-age interaction variants for pulse pressure (using ENCODE DHS annotations) and found a significant enrichment of blood vessel cells among the results. Strikingly, when applying the method to 26 complimentary pulse pressure variants without genetic-by-age interaction (i.e., variants with genome-wide significant marginal association on pulse pressure, but no GxAge), we could not identify an enrichment of blood vessel cell lines. This discrimination was consistent with comparative analyses of GxAge vs. no-GxAge variants by FUMA and DEPICT (which have also been added to the manuscript based on one comment by reviewer 1).*

*We describe the FORGE2 results in the main paper as follows (p 12):*

*This distinction was confirmed by direct variant-to-tissue mapping based om chromatin activity (FORGE2 [14]), which yielded a significant enrichment of blood vessel cells for the 26 genetic-by-age interaction pulse pressure loci (4 out of 14 blood vessel cells were mapped with nominal significance, binomial enrichment P= 0.004) but no enrichment for the 26 loci without genetic-by-age interaction (****Table S11****).*

*We have added the detailed results to the new* ***Table S11****:*

4. GWAS tissue enrichment analysis should be reported not just for pulse pressure, but across all traits with associations (not necessary for the main figure set, but some follow up analysis across traits should at least be shown in the supplement).

***Response:*** *While we acknowledge the reviewer’s suggestion as valuable, it’s important to note that a substantial number of loci (26) were identified specifically for pulse pressure, allowing for tissue or FORGE2 enrichment analyses. Unfortunately, only up to 6 loci were identified for the other traits, which fell short for meaningful enrichment analyses. Consequently, we opted not to include those results in the paper. However, we did expand our gene prioritization using FUMA from the initial 26 GxAge variants associated with pulse pressure to encompass all 44 GxAge variants. These additional results are presented in the main paper (p 8).*

*Gene prioritization based on DEPICT [9] and FUMA [10] highlighted 83 genes at these loci (****Table S4****). Clearest evidence was found for 11 genes that were prioritized by both methods at genetic-by-age interaction loci for pulse pressure: CDH13, CDKN1A, DOT1L, EFEMP1, FBN1, FBXO32, IGFBP3, KIAA1462, MRC2, SPSB1, TCF7L1.*

*The 83 prioritized genes are shown in the updated gene prioritization* ***Table S4****.*

5. analysis code should be put on a github repository or in zenodo (ideally with a short tutorial)

***Response:*** *Yes, this is important. We have already prepared a github repository with code to apply the three approaches to the regenie output. We will make the repository public with the publication of the paper.*

Minor comments:

line 160: "describe the results in the following." should be "describe the results in the following section."

***Response:*** *Thank you, this was revised.*

line 330: "Alzheimer" -> "Alzheimer's"

***Response:*** *Thank you, this was revised.*

**Second round of review**

**Reviewer 1**

The revised manuscript has shown considerable improvement in the clarity of its presentation of key analytical findings. Nonetheless, I have reservations regarding their responses concerning the methodology. My skepticism persists regarding the study's purported advancements in methodological innovation.

1. How the three sensitivity analyses can be used properly in practice seems unclear. What is the power of the analyses?

2. In Figure 3, the right panel looks strange. To better illustrate the study's primary method, which is the two-stage approach, could the authors consider varying 'f' while maintaining a consistent total sample size? Maybe it is unnecessary to show the other 1-sage approaches in the Figure.

3. If the GxAge interactions γ_G and β_GxAge are equivalent, as the authors stated, why are the powers of the two 1-sage approaches different?

4. The genetic-by-age interaction highlighted by this study is indeed intriguing and holds significance; however, I remain skeptical about the study's contribution to methodological innovation. The study does not seem to tackle the critical issues of avoiding cohort effects and accounting for the non-linear impact of age on trait assumptions through the development of novel methodologies. Moreover, the methodology presented is not as streamlined as it could be. If the focus is specifically on the genetic-by-age interaction, the initial selection of variants based on a marginal model might be deemed unnecessary.

**Reviewer 2**

No further comments.

**Authors Response**

**Point-by-point responses to the reviewers’ comments:**

# **Reviewer #1:**

The revised manuscript has shown considerable improvement in the clarity of its presentation of key analytical findings. Nonetheless, I have reservations regarding their responses concerning the methodology. My skepticism persists regarding the study's purported advancements in methodological innovation.

1. How the three sensitivity analyses can be used properly in practice seems unclear. What is the power of the analyses?

***Response:***

*We apologize that this was unclear and not fully consistent in the argument. We agree that the power of adjusting for birthyear additionally to age is very limited given the high correlation between birthyear and age in the UKB dataset. Of note, the analysis of each variant’s association with age is very helpful and well-powered. We now state results on this power computation.*

*We would like to thank the reviewer for this comment based on which, we have clearly re-thought and revised the part on the confounding by birth cohort effect and sensitivity analyses (please see more details also in our answer to comment #4).*

*In terms of sensitivity analyses, we have added a new chapter to the* ***Results*** *(page 10):*

## “Sensitivity analyses for identified 44 genetic-by-age interaction variants

*We explored potential confounding by birth cohort effect for the identified genetic-by-age interaction effects on the respective trait. While this can be explored by adjusting for birthyear, genetic-by-birthyear or age-by-birthyear interaction in theory, this is often impossible in practice when age and birthyear are highly correlated (collinearity). This is the case in UKB (r=-0.99;* ***Methods****). For the 44 variants identified with genetic-by-age interaction, we attempted adjusting for birthyear and found no impact on genetic-by-age interaction effects, but this was not surprising as this adjustment just reflected the adjustment for age; we also evaluated the genetic variant association with birthyear, but again this was expected to be equivalent to the association with age (****Table S7****).*

*Confounding of the genetic-by-age interaction by birthyear as well as age-dependent selection or survival would imply an association of the genetic variant with age. Among the 44 identified variants, we found only the APOE variant rs429358 associated with age (P_G🡪Age_=4.6x10^-5^,* ***Table S7****). Since the rs429358 allele that is less frequent in older age (C allele) is well-known for increasing risk of Alzheimer [11] and early mortality [12], it is perceivable that this variant’s association with age is explained by decreased study participation among Alzheimer patients (selection) or decreased survival rather than a birth cohort effect. While the observed APOE genetic-by-age interaction on BMI, weight and triglycerides is adjusted for age (i.e. removing age-dependent selection or survival effects at least in part), there might be residual confounding. Our sensitivity analyses to identify genetic variant association with age were reasonably powered (>95% to identify APOE effect on age of 0.12 years/allele; minimal detectable effect size at 80% power =0.09 years per allele, explains ~0.005% of the age variance; N=350,000, alpha=0.05/44, AF = 30%).”*

*Additionally, we have revised the* ***Methods*** *as follows (page 22):*

## “Sensitivity analyses to evaluate birth cohort, selection and survival effects

*A genetic-by-age interaction (GxAge) effect on the trait Y can be confounded by a covariate C that is (i) correlated with GxAge and associated with Y (explored by adjusting for C), (ii) correlated with age and exerting a genetic-by-covariate interaction (GxC) with the trait (explored by adjusting for GxC), or (iii) correlated with G and the covariate-by-age interaction (CxAge) being associated with the trait (explored by adjusting for CxAge) [7]. Birthyear can be such a confounder (birth cohort effect) In theory, this can be explored by sensitivity analyses adjusting for birthyear, GxBirthyear and AgexBirthyear. Yet, in practice, age and birthyear are often highly correlated in cohort studies leading to collinearity and thus inconclusive results for such sensitivity analyses. We explored this correlation and sensitivity analysis adjusting for birthyear:*

$$Y=\beta_{0}+\beta_{G}G+\beta_{Age}AGE+\beta_{GxAge}G\cdot AGE+\beta_{BY}BY+\beta_{C}C+\varepsilon$$

*When birthyear and AGE are highly correlated and birthyear affects G, the genetic variant G will be associated with AGE: this can be tested in practice by fitting a linear regression model with AGE as outcome,*

$$AGE=\delta_{0}+\delta_{G}G+\delta_{C}C+\epsilon$$

*An association of G with age is not only possible due to a birthyear effect, but can also result from survival or selection effects. We tested each variant identified with GxAGE for association with AGE with this model. “*

*Finally, we have revised the* ***Discussion*** *as follows (page 16):*

*“The assumptions can be compromised by birth cohort effects as well as age-dependent selection or survival. While birthyear effects can be adjusted for, in theory, it is almost impossible to dissect birthyear effects from age effects in practice when birthyear and age are highly correlated. This is the case in UKB due to the short time span of recruiting.” …. “An indicator for birthyear, survival or selection effects on a genetic variant is the genetic variant’s effect on age. We found none of the identified 44 variants associated with age, except for the APOE variant which is known for association with Alzheimer’s and early mortality [13–15]. The APOE variant’s association with age may thus be explained by age-dependent selection or survival rather than a birthyear effect.“*

2. In Figure 3, the right panel looks strange. To better illustrate the study's primary method, which is the two-stage approach, could the authors consider varying 'f' while maintaining a consistent total sample size? Maybe it is unnecessary to show the other 1-stage approaches in the Figure.

***Response:*** *We thank for the constructive comment and revised Figure 3 accordingly. We agree this figure is clearer keeping total sample size constant. Yet, we still want to include the 1-stage approaches to allow direct comparison with the 2-stage approach.*

*The updated figure looks as follows:*

*Additionally, we describe* ***the corresponding*** *results as follows (page 7):*

*“**This UKB scenario with the proportion of longitudinal data relative to total sample size (f) being only up to 15% is rather unique. To generalize to studies with higher f, we compared the power of the three approaches for varying f while keeping the total sample size constant (N_total_=N_cross_+N_long_, trait-specific) and keeping the annual change effects constant (at 10% of a medium marginal genetic effect). We expected a “kipping point” for f where the “1-stage GxAge” approach lost superiority in power against the “1-stage Change” approach. We observed trait-dependent kipping points at f=25%, 75% and 65% for BMI, LDL-C, or pulse pressure (****Figure 3****,* ***Figure S1****). For UKB sample size, power of the “1-stage Change” approach was deprecated compared to genetic-by-age interaction approaches for all traits (red dotted lines in* ***Figure 3*** *and* ***Figure S1****).”*

3. If the GxAge interactions γ_G and β_GxAge are equivalent, as the authors stated, why are the powers of the two 1-stage approaches different?

***Response:*** *We appreciate the reviewer’s question, based on which we have conducted additional analyses and investigations. It is indeed counterintuitive, at first glance, that power of the “1-stage GxAge” (in cross-sectional data) is larger than the power of the “1-stage Change” (in longitudinal data) when the number of individuals are the same in the cross-sectional and in the longitudinal data (f=50% in the right panel of new* ***Figure 3****). The longitudinal data has twice as many trait measurements as the cross-sectional data and one would expect larger power. Based on the comment, we investigated this further. First, we looked at the scenario of f=0.5 in the new* ***Figure 3*** *and described the observation in results as follows (page 7):*

*“**Interestingly, and a bit counterintuitive at first glance, equal sample size for cross-sectional and longitudinal data (f=0.5, N_cross_=N_long_=N, number of measurements N and 2*N, respectively) yielded superior power for the cross-sectional “1-stage GxAge” approach compared to longitudinal “1-stage Change” approach for blood pressure and lipid traits (e.g., for pulse pressure 85% versus 59%, for LDL-C 82% versus 12%), but reduced power for obesity traits (e.g., for BMI 92% versus 99%;* ***Figure 3, Figure S1****). This trait-dependent pattern was observed despite, across traits, the same significance level, similar sample size, and similar genetic-by-age interaction effect size relative to the phenotypic variance (i.e., similar outcome variance explained by the genetic-by-age interaction, R^2^_GxAge_ of ~0.01%,* ***Table 2****). However, a driving factor for power in longitudinal analyses is also the variance of annual outcome change explained by the genetic effect (R^2^_Change_). Indeed, we observed a smaller R^2^_Change_ for pulse pressure and LDL-C (0.009% and 0.004%, respectively) compared to BMI and weight (R^2^_Change_ ~0.04% for both traits;* ***Table 2****).”*

*Thus, the difference in power in longitudinal analyses across traits in Figure 3 at f=0.5 stems from trait-dependent R^2^_Change ._ We explored this further using a more controlled setting: we explore when the power of the genetic-by-age interaction test is equivalent to the power of testing a genetic variant for association with trait change (novel supplementary* ***Note S3*** *). We also compare aspects like trait variance and correlation, which drive power, in the dataset,* *(novel* ***Table S2****). The power of the annual trait change analyses is limited in the UKB longitudinal data due to the short follow-up time and depends on the trait due to the correlation of trait measurements at timepoint 1 and trait measurements at timepoint 2. The genetic-by-age interaction analyses can make use of the large age range (and thus large age variance).*

*We summarize the results of these theoretical considerations and observations of power computations under a controlled setting in* ***Results*** *(page 8):*

*“**To explore the reasons for these trait-dependent power differences further, we compared power of a genetic-by-age interaction test and an annual change association test in a* *more controlled scenario (fixed parameters of N_cross_=N_long_=200K, alpha=5x10^-8^, allele frequency AF=0.3, beta_GxAge_=beta_Change_). We demonstrate that the power of the genetic-by-age interaction test is larger than* *power of the annual change test, as long as* $\left[ 2*Var\left( Age \right) \right]>\left[ {age}_{diff}^{2}/(1-r_{Y1,Y2}) \right]$ *(****Note S3****). This can be interpreted intuitively: (i) the larger the age range and thus the age variance in the cross-sectional data, the larger the power of the genetic-by-age interaction test, (ii) the longer the follow-up time (age_diff_) in the longitudinal data and the closer r_Y1,Y2_ to 1 (i.e., little technical measurement error or intra-individual variability), the larger the power of the annual change test. This explains the observed trait-specific pattern (****Table S2****): the power of the annual change test compared to the genetic-by-age interaction test (i) is superior for BMI due to relatively large follow-up time and high correlation (age_diff_~7.5 years, r_Y1,Y2_~0.91), (ii) inferior for pulse pressure due to the lower correlation (age_diff_~7.5 years, r_Y1,Y2_~0.65) and (iii) near zero for LDL-C due to shorter follow-up time and lower correlation (age_diff_~4.3 years, r_Y1,Y2_~0.62). Thus, the genetic-by-age interaction test can be particularly helpful for traits where measurement error or intra-individual variability is high and when follow-up length is short.”*

*In the new Supp* ***Note S3****, the reader can find a detailed derivation of the condition when the power of the two tests are the same (given same N, alpha, and allele frequency). In* ***Table S2****, more details can be found regarding the observed parameters in the UKB datasets.*

*By this, we have now a clear sense what drives the power in the GxAge and G-> change analyses. We thank the* *reviewer very much for the astute comment, which triggered us to dig in here more deeply.*

4. The genetic-by-age interaction highlighted by this study is indeed intriguing and holds significance; however, I remain skeptical about the study's contribution to methodological innovation. The study does not seem to tackle the critical issues of avoiding cohort effects and accounting for the non-linear impact of age on trait assumptions through the development of novel methodologies. Moreover, the methodology presented is not as streamlined as it could be. If the focus is specifically on the genetic-by-age interaction, the initial selection of variants based on a marginal model might be deemed unnecessary.

***Response****: We thank the reviewer or this critical assessment. We agree that the focus of the paper is not methodological innovation that solves the problem of cohort effects or non-linearity assumption. We have thus removed the following sentence from discussion (page 17):*

*~~Thus, our novel approach is a promising route to identify genetic loci for annual trait change in such data.~~*

*We agree that the genetic-by-age interaction part highlighted here is the relevant part and have emphasized this better now in the* ***Conclusions*** *chapter by removing the following sentence from* ***Conclusions*** *(page 18):*

*~~we propose a powerful and practical approach to utilize genetic-by-age interaction in cross-sectional data to help identify genetic association with trait change in longitudinal data. The approach is directly applicable to other traits in UKB, for which large cross-sectional but limited longitudinal data is available. It is generalizable to other GWAS scenarios, such as GWAS meta-analysis consortia that may include an abundance of cross-sectional studies but sparse data on longitudinal studies.~~*

*We would also like to clarify that the selection of variants based on a marginal model allows to reduce the multiple testing burden and thus to increase power for the interaction test. This 2-step approach* *renders interaction power complementary to an interaction test at genome-wide significance level: The 2-step is beneficial for variants showing marginal and interaction effects, while the genome-wide test is beneficial for variants showing interaction but zero marginal effects. We have demonstrated this relationship in own work (PMID: 28749953). More generally, various 2-step approaches to support GxE identification have been described (e.g., PMIDs 23843674, 22714933, 36571162, 37425767).*

*Regarding the critical issue of the cohort effect: we have thoroughly discussed this in the team with our coauthors from the statistics department. The cohort effect is a problem in the cross-sectional data; in the longitudinal data, it is a calendar time effect that needs to be considered to be negligible. We have re-worked and clarified the assumptions necessary at each step of the argument in Supp* ***Note S1****. Thus, the pre-screen on GxAGE can be affected by birth cohort effect, but the validation step in the longitudinal data is not. But of course, we need to consider the calendar time effect negligible.*

*We fully agree with the reviewer that the birth cohort effect is, so far, under-acknowledged in the GWAS literature. Regarding the linearity, we have acknowledged this more clearly that we did not evaluate this further.*

*We also agree that the methodology was not presented as streamlined as it should be. We have been, indeed, not fully rigid in notation and in the presentation of the arguments. We have corrected this and have now re-written particularly the Supp* ***Note S1*** *and methods part. We have now presented much more clearly what the specific assumptions were at each step of the argument.*

*In the* ***Abstract****, we have replaced “under the assumption of no cohort effect” by “under certain assumptions” since no cohort effect is actually just one assumption.*

*Similarly, in the* ***Background*** *(page 3), we have replaced “under the assumption that there is no cohort effect” by “under certain assumptions”.*

*In the* ***Results*** *chapter on the equivalence, we clearly state these assumptions as follows (page 5):*

*“Under the assumption that t_1_ is a random time point and does not mark an intervention (observational data), that there is no calendar time effect on Y, no birth cohort effect on G and Y, that the trait changes linearly over age, and that the covariate effects are independent of age, we demonstrate the equivalence* $\hat{\gamma}_{G}$ *~* $\hat{\beta}_{GxAge}$ *(****Figure 1****,* ***Note S1****).”*

*In* ***Figure 1****, we have added a list of the five assumptions:*

*We want to again thank the reviewer for the critical feedback, based on which we were now able to improve the methodological argument.*

**Third round of review**

**Reviewer 1**

I have no further comments on this manuscript. Thank you for your efforts in addressing my previous feedback.

All of my comments have been addressed. This reviewer has no further comments.
